# Cholinesterase Inhibitors from an Endophytic Fungus *Aspergillus niveus* Fv-er401: Metabolomics, Isolation and Molecular Docking

**DOI:** 10.3390/molecules28062559

**Published:** 2023-03-11

**Authors:** Ahmed A. Hamed, Riham A. El-Shiekh, Osama G. Mohamed, Elsayed A. Aboutabl, Fify I. Fathy, Ghada A. Fawzy, Areej M. Al-Taweel, Tarek R. Elsayed, Ashootosh Tripathi, Ahmed A. Al-Karmalawy

**Affiliations:** 1 Pharmacognosy Department, Faculty of Pharmacy, Cairo University, Kasr el Aini St., Cairo 11562, Egypt; 2 Natural Products Discovery Core, Life Sciences Institute, University of Michigan, Ann Arbor, MI 48109, USA; 3 Department of Pharmacognosy, College of Pharmacy, King Saud University, Riyadh 11495, Saudi Arabia; 4 Agricultural Microbiology Department, Faculty of Agriculture, Cairo University, Giza 12613, Egypt; 5 Department of Medicinal Chemistry, College of Pharmacy, University of Michigan, Ann Arbor, MI 48109, USA; 6 Pharmaceutical Chemistry Department, Faculty of Pharmacy, Ahram Canadian University, 6th of October City 12566, Egypt

**Keywords:** endophytes, *Aspergillus niveus* Fv-er401, *Foeniculum vulgare*, asterriquinones, citrinin, molecular networking, Alzheimer’s, molecular docking

## Abstract

Alzheimer’s disease poses a global health concern with unmet demand requiring creative approaches to discover new medications. In this study, we investigated the chemical composition and the anticholinesterase activity of *Aspergillus niveus* Fv-er401 isolated from *Foeniculum vulgare* (Apiaceae) roots. Fifty-eight metabolites were identified using UHPLC-MS/MS analysis of the crude extract. The fungal extract showed acetylcholinesterase (AChE) and butyrylcholinesterase (BuChE) inhibitory effects with IC_50_ 53.44 ± 1.57 and 48.46 ± 0.41 µg/mL, respectively. Two known metabolites were isolated, terrequinone A and citrinin, showing moderate AChE and BuChE inhibitory activity using the Ellman’s method (IC_50_ = 11.10 ± 0.38 µg/mL and 5.06 ± 0.15 µg/mL, respectively for AChE, and IC_50_ 15.63 ± 1.27 µg/mL and 8.02 ± 0.08 µg/mL, respectively for BuChE). As evidenced by molecular docking, the isolated compounds and other structurally related metabolites identified by molecular networking had the required structural features for AChE and BuChE inhibition. Where varioxiranol G (−9.76 and −10.36 kcal/mol), penicitrinol B (−9.50 and −8.02 kcal/mol), dicitrinol A (−8.53 and −7.98 kcal/mol) and asterriquinone CT5 (−8.02 and −8.25 kcal/mol) showed better binding scores as AChE and BuChE inhibitors than the co-crystallized inhibitor (between −7.89 and 7.82 kcal/mol) making them promising candidates for the development of new drugs to treat Alzheimer’s.

## 1. Introduction

Alzheimer’s disease (AD), the most prevalent type of dementia in the elderly, is a neurodegenerative condition that progressively damages the patient of cognitive function and ultimately causes death [1,2]. There could be as more than 5.8 million people in the United States with Alzheimer’s disease in the year 2020. This number is expected to triple nearly by 2060 when it reaches 14 million [3]. The treatment for Alzheimer’s was expected to cost between USD 159 and USD 215 billion in 2010. By 2040, these costs are expected to rise to USD 379 billion and more than USD 500 billion annually [4]. In Alzheimer’s disease, the loss of cholinergic neurons and, consequently, the lack of acetylcholine (Ach) are directly linked to cognition decline [5]. Cholinesterase inhibitors are chemical substances that impede the activity of cholinesterases, AchE and BuChE. They stop the breakdown of the neurotransmitters acetylcholine (Ach) and butyrylcholine, improving their level in the body (brain, blood and nerve tissue) [6]. Considering its importance in cholinergic system modulation, acetylcholinesterase (AchE) has become a prime therapeutic target in the treatment of Alzheimer’s disease (AD) [7,8,9]. AchE and BuChE, two types of cholinesterase, are widespread in the body and hydrolyze the neurotransmitter acetylcholine (Ach) in the normal brain. Dual cholinesterase inhibitors targeting AchE and BuChE can balance cholinergic neurotransmission and improve memory and cognition in AD patients [10,11]. ChEIs improve cognition and behavior in some patients with AD, including tacrine, rivastigmine, donepezil and galantamine. However, they have several side effects, such as bradycardia, nausea, stomach pain, anorexia and diarrhea [12].

Among the most promising sources of novel candidates are endophytic fungi, as evidenced by the isolation of huperzine-A generating fungi from *Huperzia serrata* and nectriapyrone and tryptophol from endophytic fungus *Phomopsis* sp. [13,14,15]. Endophytic fungi are one of the most potential sources of novel active natural products [16]. They colonize the interior tissues of higher plants without harming the host [17]. In this relationship, endophytes have a wide range of bioactive secondary metabolites that can change how the host plant works, how it defends itself and how well it can handle biotic and abiotic stresses [18]. Due to their unusual chemical structures, numerous endophytic secondary metabolites have been investigated for potential use in developing new therapeutics [19]. They have also been shown as antioxidant, neuroprotective, anti-inflammatory, anticancer, antimicrobial and antiparasitic agents [20,21].

*Aspergillus* spp. Are ubiquitous fungi that produce a wide range of secondary metabolites [22,23,24,25], having a broad range of biological effects, such as cytotoxic [26] and antimicrobial activities [22,27]. *Aspergillus niveus* is reported as an endosymbiotic fungus in the digestive tract of a woodlouse from the Trichoniscidae family producing several metabolites, including citreovirdins, cytochalasans and citrinin [28,29].

However, one of the primary challenges of annotating these unique metabolites has been the requirement of isolation in appreciable quantity for structural studies. The mass spectrometry (MS) approach is sensitive, flexible and gives essential structure information that helps discover active compounds from the crude extracts. The online platform Global Natural Products Social Molecular Networking (GNPS) makes it possible to use various MS-based metabolomics tools, allowing researchers to analyze large data sets. GNPS also has a public spectral library and multiple tools that automatically find spectral matches [30]. In addition, the chemical space in a metabolomics experiment can be visualized with networking (GNPS) [31]. On the other hand, molecular docking studies represent the most essential and available types of computational chemistry nowadays. It helps to investigate the possible mechanism of action and target interactions for a particular drug candidate and compare it to a common reference [12,32,33].

Because there is a need to develop new anti-Alzheimer medications [34], in this study, we investigated the cholinesterase enzyme (ChE) inhibitory potential of *A. niveus* Fv-er401 crude EtOAc extract with tentative identification of its chemical composition using UHPLC-MS/MS-based molecular networking to highlight the active metabolites. In addition, two major metabolites, citrinin and terrequinone A were isolated and tested for ChE inhibitory activity. Moreover, evaluation of their structurally related metabolites for their inhibitory effect with the aid of molecular networking and molecular docking studies.

## 2. Results

### 2.1. UHPLC-MS/MS Analysis

Metabolite profiling was achieved by UHPLC-ESI-QTOF-MS/MS-based molecular networking to investigate and find promising metabolites with the desired bioactivity. In addition to a literature review, metabolites of the extracts were annotated using the SIRIUS platform and several databases such as NP Atlas and Reaxys. The analysis identified a minimum of 58 secondary metabolites within the extracts. Six of them were identified as hits by using the GNPS-based molecular network. Table S1 shows a list of annotated compounds.

**Figure 1:**
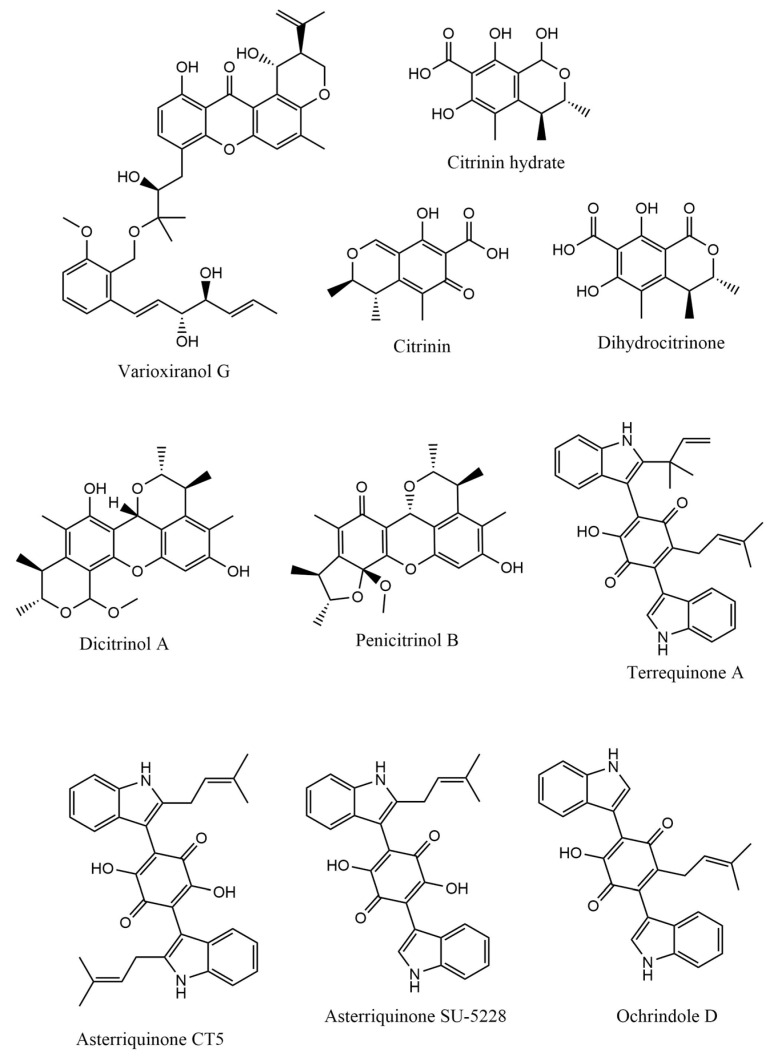
Chemical structures of selected structures of the identified metabolites.

Numerous secondary metabolites were mainly identified from *Aspergillus* and categorized into Meroterpenoids and polyketides, long-chain fatty acids and esters, sterols, sphingolipids, bis-indolyl alkaloids and citrinin analogs. Visual analysis of MS/MS data via molecular networking allows the identification of metabolites related structurally to isolated compounds with potential anticholinesterase inhibition effects. Each node correlates to one consensus MS/MS spectrum within the network, expressing precursor ion mass (*m*/*z*). Nodes displaying similar fragmentation spectra are connected with edges. The node size expresses the precursor ion intensity, whereas the edge label represents the cosine score. For the fungal isolate under investigation, two molecular networks were shown separately in positive (Figure S2) and negative (Figure S3) ionization modes. Table 1 shows a list of isolated compounds (terrequinone A and citrinin) and their putatively identified structurally related metabolites identified in fungal ethyl acetate extract, while (Figure 1) shows their structures.

**Figure 2:**
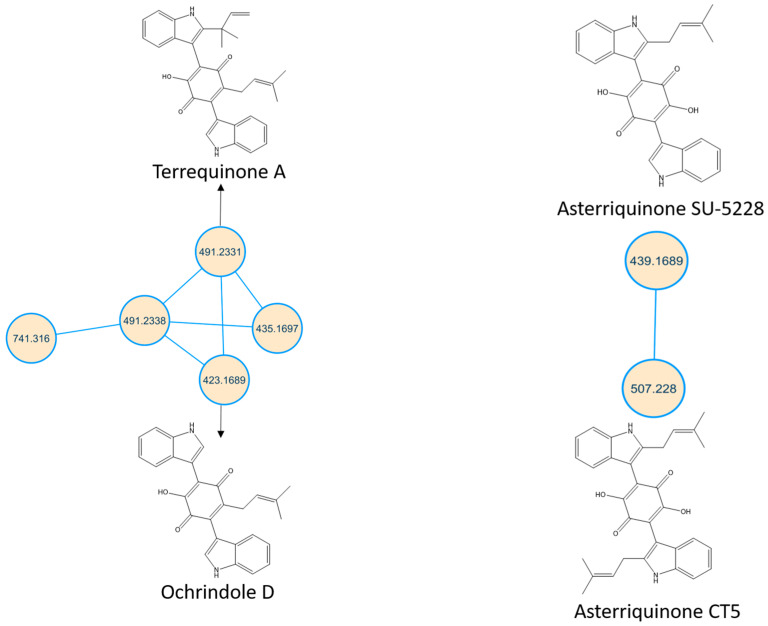
Terrequinone A and asterriquinones spectral families. The nodes label represents precursor mass (*m*/*z*).

Terrequinone A and clustered related metabolites are shown in Figure 2.

From our data, the molecular ion peak of [M+H]^+^ at *m*/*z* 491.2331 matched terrequinone A (Compound **7**). In terms of structure, compound **7** is a bis-indolyl alkaloid that contains two indole moieties combined with a central monohydroxylated quinone moiety along with two alkyl side chains that are prenyl group and tertiary pentenyl group. Key fragments such as 435.1661, 366.0949, 349.0917, 158.0538 and 69.0651 represented the loss of propylene along with vinyl groups, the loss of tertiary pentenyl and isobutylene fragments, sequential loss of the hydroxyl group, formation of 2-(1H-indol-3-yl)ethen-1-ol cation and formation of isoamylene stable allylic carbocation, respectively as seen in Figure S4. Compound **8** was identified as ochrindole D; it showed a molecular ion peak of [M+H]^+^ at *m*/*z* 423.1689. The mass difference of 68.0642 between terrequinone A and ochrindole D indicated the loss of one of the pentenyl groups. Key fragments such as 380.1069, 367.0984, 339.1042, 158.0505 and 69.0618 represented the loss of the propylene group, the loss of the isobutylene fragment, the loss of the pentenyl group along with the hydroxyl group, formation of 2-(1H-indol-3-yl)ethen-1-ol cation and formation of isoamylene stable allylic carbocation respectively, as seen in Figure S5. Compound **9** was identified as asterriquinone CT5, and compound **6** was identified as asterriquinone SU-5228, where both of them showed molecular ion peaks of [M+H]^+^ at *m*/*z* 507.228 and 439.1689, respectively. The mass difference between the two metabolites indicated the loss of one pentenyl group. Structurally, they have dihydroxylated quinone moiety rather than monohydroxylated ones in previously discussed metabolites. Compound **9** showed key fragments such as 439.1658, 371.0909, 343.0954, 327.1006, 158.0470, 69.0599, which represented the loss of one pentenyl group, the loss of two pentenyl groups, sequential loss of one of the oxo groups, sequential loss of hydroxyl group, formation of 2-(1H-indol-3-yl)ethen-1-ol cation and formation of isoamylene stable allylic carbocation respectively, as seen in Figure S6. Compound **6** showed a similar fragmentation pattern, as seen in Figure S7. Notably, terrequinone A and all related identified metabolites show fragment peaks of *m*/*z* 158 that might be characteristic of this family of metabolites. Moreover, the presence of *m*/*z* 69 suggested the presence of the prenyl group rather than the tertiary pentenyl group due to the formation of a stable allylic carbocation. Due to the difference in central quinone moiety, terrequinone A and ochrindole D showed fragment peaks at *m*/*z* 339.1042, while asterriquinone CT5 and asterriquinone SU-5228 showed fragment peaks at *m*/*z* 343.0954 and 327.1006.

**Figure 3:**
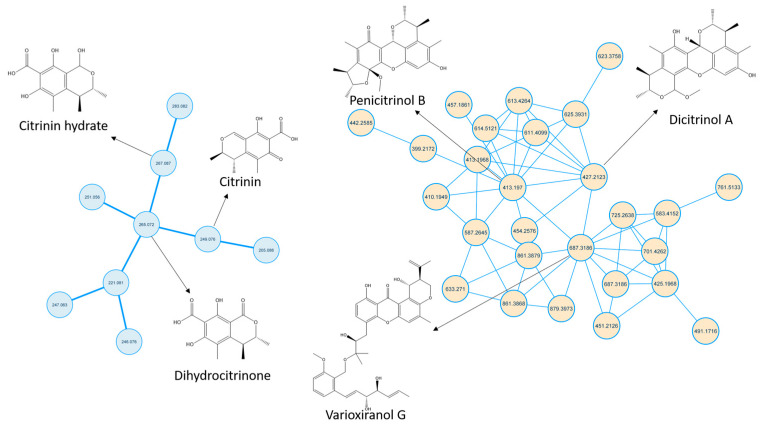
Citrinin, citrinin dimers and related metabolites spectral families. The node label represents precursor mass (*m*/*z*).

The molecular Ion peak of [M-H]- at 249.0802 *m*/*z* matched citrinin (Compound **3**). Citrinin, along with related metabolites, is shown in Figure 3.

Key fragments 231.0664, 205.0869, 177.0920 and 161.0974 represented the loss of the hydroxyl group, carboxylic group, loss of oxo group with carboxylic group and sequential loss of the hydroxyl group, respectively, as shown in Figure S8.

Compound **2** was identified as citrinin hydrate, and Compound **1** was identified as dihydrocitrinone, where both showed molecular ion peaks of [M-H]^-^ at *m*/*z* 267.0909 and 265.0733, respectively. Compound **2** showed fragments 249.0780, 231.0661, 205.0878 and 179.0713, representing the loss of one hydroxyl group, two hydroxyl groups, one hydroxyl group along with the carboxylic group and sequential loss of two methyl groups, respectively, as shown in Figure S9. Compound **1** showed fragments 247.0611, 221.0818 and 177.0919 represented the loss of the hydroxyl group, the loss of the carboxylic group and the sequential loss of pyranone COO, respectively, as shown in Figure S10. Notably, citrinin and all related identified metabolites show a fragment peak of *m*/*z* 79, corresponding to the formation of penta-1,4-dien-2-ol carbanion. This fragment might be characteristic of this family of metabolites.

Compound **4** was identified as dicitrinol A, and compound **5** was identified as penicitrinol B, where both of them showed molecular ion peaks of [M+H]^+^ at *m*/*z* 427.2123 and 413.197, respectively. They belong to a class of metabolites called citrinin dimers that are structurally related to citrinin. They showed similar fragmentation patterns due to close structure similarity, as seen in Figures S11 and S12. However, the presence of *m*/*z* 95.0788 in compound **5** indicated the presence of the 2,3-dimethylfuran group, while absent in compound **4**. Moreover, the presence of fragment *m*/*z* 233.0817 in compound **4** further supported the existence of benzopyran rather than benzofuran moiety. Compound **10** was identified as varioxiranol G. it showed a molecular ion peak of [M+H]^+^ at *m*/*z* 687.3186. Structurally, varioxiranol G comprises an uncommon combination of xanthone moiety with benzyl alcohol via an ether bond [28]. Key fragments such as 425.1972, 219.1025, 207.1014 and 69.0690 represented the loss of benzyl alcohol moiety, loss of alkyl xanthone moiety with vinyl group loss, loss of benzyl alcohol moiety along with xanthone ring cleavage and formation of 2-beuten-1-ol cation, respectively (Figure S13).

### 2.2. Isolation

Consequently, two major constituents in the extract were isolated and identified terrequinone A and citrinin. The isolated compound, identified as terrequinone A, was obtained before from rhizosphere fungi of Sonoran Desert *Aspergillus terreus*. It was reported to have moderate cytotoxic activity against NCI-H460, MCF-7, SF-268, MIA Pa Ca-2 cell lines with IC_50_ values of 5.60 µM, 6.80 µM, 13.90 µM and 5.40 µM, respectively [35]. Another isolated compound, citrinin, was recovered from the extract of a strain of *A. niveus* isolated from barley [36]. Citrinin belongs to a class of metabolites called benzopyrans [37].

### 2.3. Cholinesterase Inhibitory Activity Studies

The crude extract of *A. niveus* Fv-er401 was tested against AChE and BuChE, revealing dual enzyme inhibition where IC_50_ is shown in Table 2, where the isolated compounds had moderate activity as anti-acetyl cholinesterase with IC_50_ of 22.62 ± 0.77 μM for terrequinone A and 20.12 ± 0.59 μM for citrinin. In addition to anti-butyryl cholinesterase activity with IC_50_ of 31.85 ± 2.58 μM and 30.12 ± 0.4 μM, respectively (Table 2).

### 2.4. Docking Study

Computational studies on the cholinesterase inhibitory activities toward the discovery of novel neurotherapeutics were assessed in our study. The isolated compounds and their structurally related identified metabolites were subjected to two molecular docking studies to investigate their potential inhibitory effects against both AChE and BuChE receptors. Donepezil, together with the co-crystallized inhibitors of the AChE and BuChE receptors, were used as reference standards.

Analyzing the binding pocket of AChE (PDB ID: 1OCE), it was clear that the co-crystallized inhibitor bound the following amino acids; Ser200 (covalent bond), Ala201, Gly118 and Gly119 (hydrogen bonds). On the other hand, the co-crystallized inhibitor of BuChE (PDB ID: 7BO4) stabilized within its binding pocket by forming hydrogen bonds with Asp70, Ser198 and Leu286 amino acids. Moreover, it formed pi-hydrogen bonds with Gly117 and Phe329 amino acids. This indicates the great importance of the aforementioned amino acids in inducing the antagonistic activities of both the AChE and BuChE co-crystallized inhibitors.

Studying the docking results of all derivatives and correlating them with the obtained biological data, it was clear that bis-indolyl alkaloids, citrinin analogs, citrinin dimers and varioxiranol G were found to be promising. Their names, docking scores and RMSD values are represented in Table 3 and compared to donepezil and the co-crystallized inhibitors of AChE and BuChE receptors. Based on the docking scores and biological data values, we selected dicitrinol A, terrequinone A, varioxiranol G and asterriquinone CT5 for further investigations.

First, donepezil formed one pi-hydrogen interaction with the Gly118 amino acid of the AChE binding pocket. Moreover, it showed the formation of one pi-hydrogen interaction with the Phe329 amino acid of the BuChE binding site. However, the docked co-crystallized ligand of AChE showed the formation of three hydrogen bonds with Asp72, Gly118 and Gly119 and one hydrogen-pi interaction with Phe330 amino acids. Besides, the docked co-crystallized ligand of BuChE got stabilized within its binding pocket by forming one ionic bond with Asp70, one hydrogen bond with Leu286 and two pi-hydrogen interactions with Phe329 amino acids, Table S4.

On the other hand, dicitrinol A showed the formation of two hydrogen bonds with Trp84 and Phe330 and one pi-H interaction with Gly118 amino acids of the AChE receptor. Besides, it formed only one hydrogen bond with the Gly116 amino acid of the BuChE receptor. Moreover, terrequinone A bound Trp84 and Gly118 amino acids with two pi-hydrogen bonds, and Tyr334 amino acid with a pi-pi bond within the AChE binding pocket. Furthermore, it formed one hydrogen-pi bond to Tyr332 and two pi-pi bonds with Trp82 amino acids of the BuChE receptor. For varioxiranol G, it was obvious that two hydrogen bonds were formed to Asp285 and Ser286 and one pi-hydrogen bond to Tyr334 amino acids of the AChE. Whereas within the BuChE, it bound Ser198 amino acid with a hydrogen bond and Pro285 amino acid with a pi-hydrogen bond. Moreover, asterriquinone CT5 formed two pi-hydrogen interactions with Asp72 and Tyr334 amino acids and one pi-pi interaction with Tyr334 amino acid as well as the AChE. However, it formed two hydrogen bonds to Asp70 and Ser287 amino acids of the BuChE binding site, as depicted in Table 4. Based on the previously discussed results, we can conclude the greatly recommended inhibitory activities of the examined candidates against both the AChE and BuChE receptors. This can be confirmed through their very promising binding scores, which exceeded -in some cases- those of the co-crystallized inhibitors of the AChE and BuChE.

## 3. Discussion

Endophytes produce numerous unique biologically active metabolites [38,39]. Despite their extraordinary potential as producers of bioactive natural compounds, there are limited reports on endophytic fungi’s cholinesterase inhibitory action. The genus *Aspergillus* has proven to be a rich source of bioactive compounds [22,23,24]. In our study, the endophytic fungi were isolated from *F. vulgare* (Apiaceae), as it is rich in bioactive therapeutic molecules.

*Aspergillus* metabolites exhibited various biological properties, including antioxidant and neuroprotective effects [40,41,42]. In our study, *A. niveus* extract showed promising anticholinesterase activity. Moreover, we isolated and characterized two metabolites previously reported from the genus *Aspergillus* and accomplished mass-spectrometry-based identification of fifty-eight metabolites. Additionally, we reported the anticholinesterase activities of isolated metabolites (terrequinone A and citrinin) as well as the fungal extract. This study investigated the anticholinesterase activities of citrinin and terrequinone A for the first time. Further, structurally related metabolites identified by molecular networking were assessed with molecular docking.

Asterriquinones could potentially treat neurodegenerative diseases such as Alzheimer’s and Parkinson’s by activating tyrosine kinases (TrkA, TrkB and TrkC) receptors [43,44,45]. Demethylasterriquinone-B1 analogs showed promising neuroprotective activity [46]. The study revealed that indolylquinone is a key pharmacophore that can activate the nerve growth factor receptor, which belongs to a class of neurotrophin proteins and supports neuronal growth and survival [46]. In 2004, a novel metabolite, terrequinone A, was isolated from the EtOAc extracts of a fungus living in the roots of plants in the Sonoran Desert. The data revealed that the central quinone moiety is monohydroxylated compared to all known asterriquinones bearing dihydroxylated quinone moieties [35]. Since terrequinone A is similar in structure to demethylasterriquinone-B1, it could possess similar mechanisms in neuroprotection action as potential neurotrophin mimics. Supported by the aforementioned study, the promising bioassay results of terrequinone A and binding scores revealed by molecular docking of asterriquinones identified in the fungal extract, asterriquinones should be considered promising candidates for the development of Anti-Alzheimer’s medications.

Citrinins were previously reported as potential neuroprotective agents, and their ability to cross the blood-brain barrier supports their actions [47,48]. Citrinin showed a potential neuroprotective activity at low doses against glutamate-induced excitotoxicity [49]. Unfortunately, compounds of this class greatly concern potential long-term toxicity impeding further development. The toxicity greatly depends on exposure time, concentration, frequency and route [48]. To surmount this obstacle, synthesizing more analogs is of great importance.

Furthermore, our study is the first report to highlight the anticholinesterase activity of citrinin dimers where dicitrinol A and penicitrinol b are assessed along with varioxiranol G with the aid of molecular docking. They showed better binding scores as AChE and BuChE inhibitors than the co-crystallized inhibitor. Isolation of these metabolites, structural characterization and bioassay are required to ensure their potentiality as anticholinesterase agents.

Overall, for the first time, this study highlights the extensive chemical investigation of *A. niveus* extract and its potential use as a cholinesterase inhibitor. Additionally, we further investigated the anticholinesterase activity of the secondary metabolites via enzyme assay and molecular docking studies. Future studies are needed to establish the clinical relevance of such metabolites and to determine a valid correlation with our in vitro efficacy results, which could be followed by in vivo testing in animal models of AD. Structural modifications could improve pharmacokinetics, pharmacodynamics and structure-activity relationship analyses in future research. Synergistic effects within these compounds and between reported drugs should be further studied to discover the mechanism beyond the anti-Alzheimer’s activity of these metabolites. More research is needed, particularly in vivo investigations and toxicity studies, before these metabolites can be classified as biomedical agents.

## 4. Materials and Methods

### 4.1. Molecular Identification of Endophytic Fungi

The fungus was isolated from the healthy *Foeniculum vulgare* (Apiaceae) roots collected from the Experimental Plants Station at Cairo University Faculty of Pharmacy. Endophytic fungi were isolated from *F. vulgare* using the method reported by Radji et al. (2011) [50]. In brief, root samples were washed for 10 min under running tap water and then dried in the air. Before surface sterilization, roots were cut into one cm-long pieces using sterile surgical blades. Surface sterilization was accomplished by immersing the sample pieces in 70% ethanol for 1 min, 5.25% sodium hypochlorite solution for 5 min, 70% ethanol for 30 s and sterile distilled water for 3–5 min in that order. Sliced pieces were placed on Petri-dishes containing potato dextrose agar (PDA) supplemented with Amoxicillin at 50 mg/L to limit bacterial growth and incubated at 28 °C until the outgrowth of endophytic fungi could be observed. Then, the pure culture was transferred to a flask with potato dextrose medium free from antibiotics, and they were cultivated for seven days at 28 °C (for media composition, see Table S2). Genomic DNA was extracted using the GeneJET Genomic DNA purification kit (Thermo Scientific, Vinius, Lithuania). Agarose gel electrophoresis and a Nanodrop spectrophotometer were used to examine DNA yields and purity. Following the protocols of White et al. (1990) and Gardes and Bruns (1993) [51,52], the internal transcribed spacer (ITS) region was amplified using the universal primers ITS1 (5′-CTTGGTCATTTAGAGGAAGTAA-3′) and ITS4 (5′-TCCTCCGCTTATTGATATGC-3′) and the PCR products were checked via agarose gel electrophoresis then sequenced by Macrogen-Koria By comparing the sequences of the amplified ITS region from this study’s fungal isolate with the most similar hits acquired from the NCBI GenBank database, a phylogenic analysis was inferred using the Neighbor-Joining method using the Maximum Composite Likelihood [53]. Evolutionary analyses were conducted in MEGA 5 software [54]. According to BLAST analysis at the NCBI database (http://www.ncbi.nlm.nih.gov accessed on 2 March 2023), the amplified ITS sequence of strain Fv-er401 shows the highest degree of similarity (99% identical) with the species *A. niveus*. A phylogenetic tree created by MEGA 5 software. Maximum Likelihood analysis was constructed using the top similar ITS sequences shown after BLASTn on NCBI GenBank using *Aspergillus niveus* Fv-er401 ITS sequence as the query, as shown in Figure S1.

### 4.2. Scale-Up Cultivation

A seed culture of *A. niveus* was prepared by inoculating a 250 mL flask containing potato dextrose broth medium (80 mL) with a single fungus colony and shaking at 190 rpm at 26.5 °C for seven days. Aliquots of the seed culture (50 µL) were transferred to individual Wickerham agar plates (×300), which were incubated at 26.5 °C for seven days. After the incubation period, the agar was diced (1.5 cm × 1.5 cm), transferred to 2 L flasks and extracted with EtOAc (2 × 400 mL) by shaking at 150 rpm overnight. The decanted organic layer was then concentrated under a vacuum to yield a crude extract (4.5 g). (100 mg) of crude extract was kept for UHPLC-MS/MS analysis and biological evaluation.

### 4.3. LC-MS/MS Analysis

Aliquots (1 µL) of EtOAc extracts (1 mg/mL in MeOH) were subjected to ultra-high performance liquid chromatography (UHPLC) using an Agilent LC-MS system including an Agilent 1290 Infinity II UHPLC and an Agilent 6545 ESI-Q-TOF-MS applying the following conditions: a Kinetex phenyl-hexyl (1.7 μm, 2.1 × 50 mm) column, 1 min isocratic elution of 90% A (A: 100% H_2_O + 0.1% formic acid) followed by 6 min linear gradient elution to 100% B (95% MeCN + 5% H_2_O + 0.1% formic acid), 0.4 mL/min. ESI conditions were adjusted as follows: capillary temperature at 320 °C, source voltage at 3.5 kV and a sheath gas flow rate of 11 L/ min. Ions in separate positive and negative modes were detected in the full scan at an intensity above 1000 counts at 6 scans/s, with an isolation width of 1.3 ~*m*/*z*, a maximum of 9 selected precursors per cycle and using ramped collision energy (5 × *m*/*z*/100 + 10 eV). Hexakis (1H,1H,3H-tetrafluoropropoxy)-phosphazene C_18_H_18_F_24_N_3_O_6_P_3_ [M+H]^+^ ion (*m*/*z* 922.0098) and purine C_5_H_4_N_4_ [M+H]^+^ ion (*m*/*z* 121.0508) were employed as internal lock masses for positive mode while hexakis (1H,1H,3H-tetrafluoropropoxy)-phosphazene C_18_H_18_F_24_N_3_O_6_P_3_ [M+TFA−H]^−^ ion (*m*/*z* 1033.9881) and TFA C_2_HF_3_O_2_ [M−H]^−^ ion (*m*/*z* 112.985587) were employed as internal lock masses for negative mode.

### 4.4. MS/MS Data Pre-Processing

The LC-MS/MS data were converted into the mzXML format using the ProteoWizard tool Msconvert [55]. The feature-based molecular networking (FBMN) workflow on GNPS was used to develop the molecular network. Before exporting to GNPS for FBMN analysis, the mass spectrometry data were processed with MZmine 2.53. Free software MZmine 2 was used to remove noise and generate peak lists automatically (for parameters, see Table S3) [56]. Plain media Wickerham agar plates were used as a blank. Features detected in MeOH and blank media were deleted from the peak lists.

### 4.5. Molecular Networks Generation

With the cross-platform Winscp, converted mzXML files were uploaded to the GNPS server (massive.ucsd.edu). For network construction, the precursor ion mass tolerance was set to 0.02 Da, and the fragment ion mass tolerance was set to 0.02 Da. What follows is the configuration of the network’s advanced settings: maximum connected component size: 100; network TopK: 10; minimum cluster size: 2; minimum pairs cosine: 0.65; minimum matched fragment ions: 4. For the library search, a score threshold of 0.65 was used and minimum matched peaks were set to 4. All the other parameters were set to their default values. The Data were further analyzed by Spec2Vec through the GNPS web interface, where the minimum pairs score was set to 0.65. The output network was merged to FBMN and visualized using Cytoscape 3.9.1 software [57]. The negative mode data was treated the same way; however, minimum pairs cosine was set to 0.8 and minimum matched fragments were set to 2.

### 4.6. Molecular Formula Prediction and Metabolites Identification

The structural interpretation was achieved by extensively examining the high-resolution mass spectra. The software Sirius 5.6.2 was used to determine the possible molecular formula of each analyte based on the high-resolution mass [58]. Based on the accurate mass and fragmentation pattern, the most likely molecular formula of the analyte was selected and searched for in chemical structure databases. In addition to the CSI:FingerID interface of SIRIUS 5.6.2 that was employed for molecular annotation, molDiscovery [59], Reaxys [60] and Natural Products Atlas [61] were also used. The list of metabolites that matched the searched elemental composition was thoroughly examined to evaluate whether it contained compounds that could be related to fungal metabolites. All detected metabolites had a mass error of less than 10 ppm.

### 4.7. Isolation of Compounds

The ethyl acetate extract (4.4 g) was partitioned between *n*-hexane (3 × 100 mL) and MeOH (100 mL), and both fractions were concentrated in vacuo to afford *n*-hexane (1.3 g) and MeOH (3.1 g) fractions. The MeOH fraction (3.1 g) was subjected to column chromatography over silica gel (200–300 mesh, 25 × 2.5 cm) and gradient eluted with dichloromethane (DCM) as follows: MeOH mixtures at 1% increments to yield 18 fractions (Fr. 1–Fr. 18) based on TLC analysis. Fr. 2 (40 mg) and Fr. 11 (55 mg) were selected for further fractionation. Column chromatography of Fr. 2 on silica gel (230–400 mesh, 13 × 1 cm) and isocratic elution with (DCM/MeOH, 97:3) afforded compound **7** (10 mg, 0.227 %*w*/*w*), which was further purified on Sephadex LH-20 (100% MeOH). Fr. 11 (55 mg) was subjected to Sephadex LH-20 chromatographic purification (13 × 1 cm) with isocratic elution with MeOH to yield compound **3** (11 mg, 0.25 % *w*/*w*).

### 4.8. Spectral Data

Compound **7** (in LC/MS table); (terrequinone A) [35]; ^1^H NMR (400 MHz, DMSO-*d*_6_) δ 11.58 (s, 1H), 10.92 (s, 1H), 10.30 (s, 1H), 7.54 (d, 1H), 7.52 (d, J = 2.6 Hz, 1H), 7.41 (d, J = 8.1 Hz, 1H), 7.35 (d, J = 8.1 Hz, 1H), 7.26–7.18 (m, 1H), 7.18–7.06 (m, 3H), 7.00–6.91 (m, 1H), 6.11 (dd, J = 17.5, 10.5 Hz, 1H), 5.06 (dd, J = 17.5, 1.5 Hz, 1H), 5.05–4.98 (m, 1H), 4.99 (dd, J = 10.5, 1.5 Hz, 1H), 3.27 (dd, J = 13.1, 7.1 Hz, 1H), 3.16 (dd, J = 13.0, 6.9 Hz, 1H), 1.59 (s, 3H), 1.48 (d, J = 2.0 Hz, 6H), 1.29 (S, 3H).

Compound **3** (in LC/MS table); (citrinin) [62]; ^1^H NMR (400 MHz, CDCl_3_) δ 15.79 (s, 1H), 15.04 (s, 1H), 8.16 (s, 1H), 4.70 (dq, J = 30.6, 6.7 Hz, 1H), 2.91 (dq, J = 34.3, 7.3 Hz, 1H), 1.95 (s, 3H), 1.27 (d, J = 6.7 Hz, 3H), 1.16 (d, J = 7.2 Hz, 3H)**.** 13C NMR (101 MHz, CDCl_3_) δ 9.46, 18.26, 18.52, 34.61, 81.64, 100.36, 107.42, 123.13, 138.99, 162.67, 174.53, 177.21, 183.83.

### 4.9. Cholinesterase Inhibitory Activity Studies

The assays were performed following the standard method described by Srour et al. (2023) [63]. In total, 170 μL of Tris–HCl buffer (200 mM, pH 7.5) was added, then 20 μL of the tested compounds at different concentrations (50–1.5625 g mL^−1^), and finally 20 μL of the enzyme solution (0.1 U mL1). After 10 min of incubation at 25 °C, 40 μL of DTNB (dithio-bis (2-nitrobenzoic acid)) and 20 μL of the substrate (1.11 mM) were added. In BChE and AChE assays, butyrylthiocholine iodide and acetylthiocholine were used as substrates, and DTNB was used as an indicator. MeOH was used to dissolve the tested samples. Using a microplate reader, the intensity of the developed color was measured at 405 nm (Reading A) and a control without the inhibitor was measured (Reading B). Blank tests were performed by replacing the enzyme (20 μL) with buffer and recording the absorbances. This was performed to account for the fact that the indicator might break down spontaneously or the inhibitor might have a natural color. Linear regression was used to determine the IC_50_ (50% inhibitory concentration) (Figure S14). The Microsoft EXCEL 365 program and the graph pad instate 6.0 program were used for data analysis.

Percentage inhibition was calculated as follows:

$$\%\mathrm{Inhibition}=\frac{\mathrm{Reading}\;B-\mathrm{Reading}\;A}{\mathrm{Reading}\;B}\times 100$$

The IC_50_ values were calculated from the curves using Microsoft EXCEL 365 program.

### 4.10. Molecular Docking Studies

The isolated compounds and their identified derivatives were subjected to two general molecular docking studies targeting both the AChE and BuChE receptors using the MOE 2019.0102 software [64,65]. Donepezil and the co-crystallized inhibitors of the AChE and BuChE receptors were used as reference standards.

The identified isolates and donepezil were sketched individually into the ChemDraw Professional copied and pasted into the MOE working window. Each compound was corrected for its partial charges and energy minimized as described before [66,67]. All the prepared candidates were inserted into two separate databases with the presence of the co-crystallized inhibitor of the AChE and BuChE targets, respectively. Further, the X-ray structures of both AChE and BuChE receptors were downloaded from the Protein Data Bank (https://www.rcsb.org/structure/1OCE and https://www.rcsb.org/structure/7BO4 accessed on 2 March 2023, respectively, accessed on 23/12/2022). Each protein was opened in the MOE working window and prepared for docking through correction, 3D hydrogenation and energy minimization, as discussed earlier [68,69]. Finally, two docking processes were performed using the appropriate database in each case. The program specifications were adjusted as previously mentioned [70,71]. The best pose for each one of the most promising compounds was selected for further investigation. Besides, two redocking processes of the co-crystallized inhibitors of both AChE and BuChE receptors into their binding pockets were performed. This was performed to validate the MOE 2019.0102 software [72,73], which was confirmed by obtaining low root mean square deviation (RMSD) values (<2 Å). Moreover, closely similar binding modes between the docked and native co-crystallized ligands were observed.

## 5. Conclusions

This study highlighted the potential role of the endophytic fungus *A. niveus* as a source of anticholinesterase agents. The fungal extract and isolated compounds displayed moderate activity via dual inhibition effects against AChE and BuChE enzymes. With the aid of molecular networking, structurally related metabolites to terrequinone A and citrinin were identified, yielding ten metabolites with considerable anticholinesterase activities. They exhibited considerably similar binding modes to the co-crystallized ligands of the AChE and BuChE. Collectively, for the first time, we report asterriquinones, citrinin analogs, citrinin dimers and varioxiranol G as promising leads for developing new potent anticholinesterase agents.

## Figures and Tables

**Table 1:** Putatively identified unique metabolites of *A. niveus* Fv-er401 ethyl acetate extract in positive and negative modes.

No.	R_t_ (min)	Compound Name	Adduct	Precursor Mass	Molecular Formula	MS/MS Fragmentation Product Ions	Chemical Class
1	3.2701	Dihydrocitrinone	M-H	265.0733	C_13_H_14_O_6_	79.0185, 162.0682, 177.0918, 221.0819, 247.0610	Benzopyrans
2	4.0729	Citrinin hydrate	M-H	267.0909	C_13_H_16_O_6_	79.0190, 179.0714, 205.0881, 249.0783, 267.0874	Benzopyrans
3	4.166	Citrinin	M-H	249.0802	C_13_H_14_O_5_	79.0189, 161.0974, 177.0920, 205.0859, 231.0664	Benzopyrans
4	5.0989	Dicitrinol A	M+H	427.2123	C_25_H_30_O_6_	191.1066, 201.0906, 219.1021, 220.1052, 233.0817, 409.1543	Citrinin dimers
5	5.1543	Penicitrinol B	M+H	413.197	C_24_H_28_O_6_	95.0788, 191.1003, 201.0844, 219.0953, 220.0988, 395.1788	Citrinin dimers
6	5.2098	Asterriquinone SU-5228	M+H	439.1689	C_27_H_22_N_2_O_4_	69.0701, 158.06, 327.1138, 343.1087, 371.1034	Bisindole alkaloids
7	5.764	Terrequinone A	M+H	491.2331	C_32_H_30_N_2_O_3_	69.0651, 158.0538, 349.0917, 366.0949, 379.1029, 435.1661	Bisindole alkaloids
8	5.764	Ochrindole D	M+H	423.1689	C_27_H_22_N_2_O_3_	69.0618, 158.0505, 339.1042, 350.0923, 367.0984, 380.1069	Bisindole alkaloids
9	5.9579	Asterriquinone CT5	M+H	507.228	C_32_H_30_N_2_O_4_	69.0599, 158.0470, 327.1006, 343.0954, 371.0909	Bisindole alkaloids
10	6.5675	Varioxiranol G	M+H	687.3186	C_40_H_46_O_10_	69.0690, 207.1024, 219.1022, 425.1973, 493.2238	Polyketides

**Table 2:** Cholinesterase inhibitory activity studies.

	Acetylcholine Esterase		Butyryl Choline Esterase		Molecular Weight
	IC_50_ (μg/mL)	IC_50_ (μM)	IC_50_ (μg/mL)	IC_50_ (μM)	
*A. niveus* Fv-er401 EtOAc extract	53.44 ± 1.57		48.46 ± 0.41		-
Citrinin	5.06 ± 0.15	20.12 ± 0.59	8.02 ± 0.08	30.12 ± 0.4	250.25
Terrequinone A	11.10 ± 0.38	22.62 ± 0.77	15.63 ± 1.27	31.85 ± 2.58	490.603
Donepezil	0.22 ± 0.03	0.59 ± 0.083	0.29 ± 0.0033	0.77 ± 0.0088	379.492

**Table 3:** The docking scores and RMSD values for the most promising ten candidates from *A. niveus* Fv-er401 within the binding sites of both AChE and BuChE receptors. Also, donepezil and the co-crystallized inhibitors are depicted as reference standards.

No.	Compound	AChE		BuChE	
		Score (kcal/mol)	RMSD (Å)	Score (kcal/mol)	RMSD (Å)
1	Citrinin hydrate	−6.61	1.25	−6.52	1.19
2	Citrinin	−6.62	1.85	−5.91	1.42
3	Dicitrinol A	−8.53	2.17	−7.98	1.12
4	Penicitrinol B	−9.50	0.79	−8.02	0.95
5	Terrequinone A	−7.34	1.52	−8.52	1.10
6	Ochrindole D	−8.11	1.77	−7.88	1.43
7	Varioxiranol G	−9.76	1.9	−10.36	1.41
8	Dihydrocitrinone	−6.48	1.09	−6.43	1.03
9	Asterriquinone SU-5228	−8.25	1.72	−7.73	1.72
10	Asterriquinone CT5	−8.02	2.07	−8.25	1.79
11	Donepezil	−8.04	1.01	−7.94	1.31
12	Co-crystallized inhibitor	−7.89	1.93	−7.82	1.59

**Table 4:** The 2D, 3D interactions and receptor pocket positioning for the most promising candidates within the binding sites of both AChE and BuChE receptors.

Comp.	R *	2D Interactions	3D Interactions	3D Positioning
Dicitrinol A	AChE	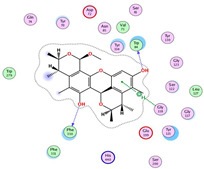	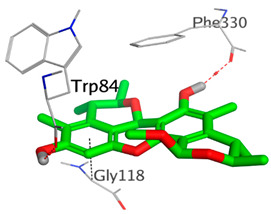	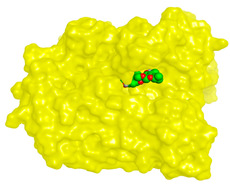
	BuChE	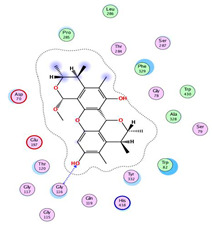	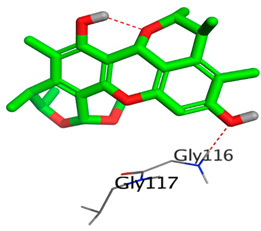	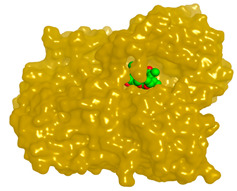
Terrequinone A	AChE	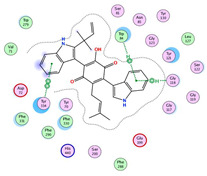	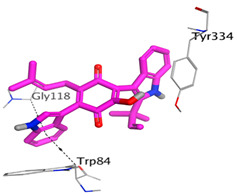	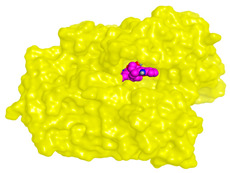
	BuChE	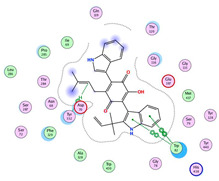	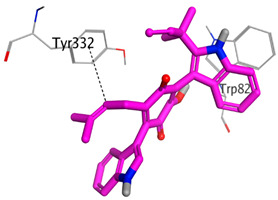	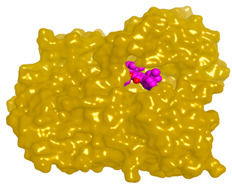
Varioxiranol G	AChE	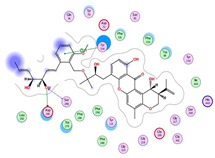	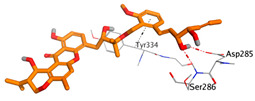	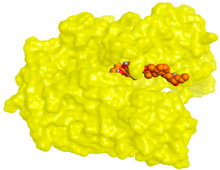
	BuChE	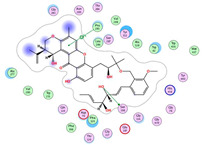	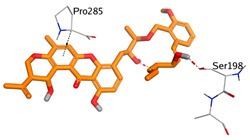	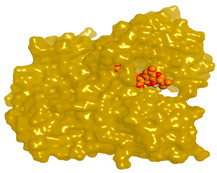
Asterriquinone CT5	AChE	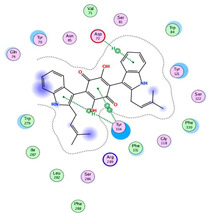	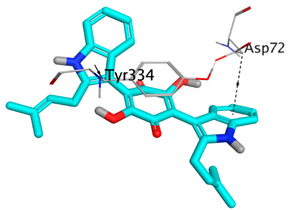	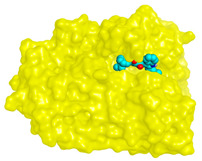
	BuChE	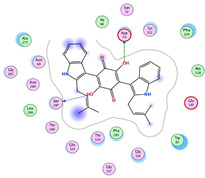	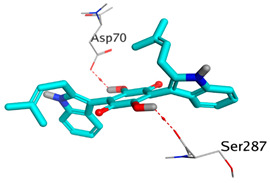	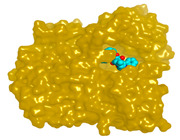

R *: Receptor.

## Data Availability

Not applicable.

## Supplementary Materials

The following supporting information can be downloaded at: https://www.mdpi.com/article/10.3390/molecules28062559/s1, Figure S1: Phylogenetic analysis of *Aspergillus niveus* Fv-er401; Figure S2: GNPS molecular network of *A. niveus* Fv-er401 ethyl acetate extract in the positive ion mode. The node label represents precursor mass (*m*/*z*), while the edge label represents the cosine score; Figure S3: GNPS molecular network of *A. niveus* Fv-er401 ethyl acetate extract in the negative ion mode. The node label represents precursor mass (*m*/*z*), while the edge label represents the cosine score; Figure S4: MS/MS fragmentation of terrequinone A (Compound **7**); Figure S5: MS/MS fragmentation of ochrindole D (Compound **8**); Figure S6: MS/MS fragmentation of asterriquinone CT5 (Compound **9**); Figure S7: Predicted fragmentation pattern of asterriquinone SU-5228 (Compound **6**); Figure S8: MS/MS fragmentation of citrinin (Compound **3**); Figure S9: MS/MS fragmentation of citrinin hydrate (Compound **2**); Figure S10: MS/MS fragmentation of dihydrocitrinone (DHC, Compound **1**); Figure S11: MS/MS fragmentation of dicitrinol A (Compound **4**); Figure S12: MS/MS fragmentation of penicitrinol B (Compound **5**); Figure S13: MS/MS fragmentation of varioxiranol G (Compound **10**); Figure S14: Graphs of acetylcholinesterase and butyrylcholinesterase inhibitory activity; Table S1: Putatively identified features of A. niveus Fv-er401 ethyl acetate extract in positive and negative modes; Table S2: Types and compositions of media; Table S3: Parameters for MZmine processing of UHPLC-MS/MS data; Table S4: 2D, 3D interactions and receptor pocket positioning for donepezil and the docked co-crystallized inhibitors within the binding sites of both AChE and BuChE receptors.
