# Supplementary material for: Cholinesterase Inhibitors from an Endophytic Fungus Aspergillus niveus Fv-er401: Metabolomics, Isolation and Molecular Docking
Source: Molecules. 2023 Mar 11;28(6):2559. doi: 10.3390/molecules28062559 (PMC10052609; doi:10.3390/molecules28062559)
Supplement: Supplementary file 1 [file molecules-28-02559-s001.zip › molecules-2240020-supplementary.pdf]

Supporting Information

Cholinesterase Inhibitors from an Endophytic Fungus *Aspergillus niveus* Fv-er401: Metabolomics, Isolation and Molecular Docking

Ahmed A. Hamed [1,*], Riham A. El-Shiekh [1], Osama G. Mohamed [1,2], Elsayed A. Aboutabl [1], Fify I. Fathy [1], Ghada A. Fawzy [1,*], Areej M. Al-Taweel [3], Tarek R. Elsayed [4], Ashootosh Tripathi [2,5] and Ahmed A. Al-Karmalawy [6]

[1] Pharmacognosy Department, Faculty of Pharmacy, Cairo University, Kasr el Aini St., Cairo 11562, Egypt
[2] Natural Products Discovery Core, Life Sciences Institute, University of Michigan, Ann Arbor, MI 48109, USA
[3] Department of Pharmacognosy, College of Pharmacy, King Saud University, Riyadh 11495, Saudi Arabia
[4] Agricultural Microbiology Department, Faculty of Agriculture, Cairo University, Giza 12613, Egypt
[5] Department of Medicinal Chemistry, College of Pharmacy, University of Michigan, Ann Arbor, MI 48109, USA
[6] Pharmaceutical Chemistry Department, Faculty of Pharmacy, Ahram Canadian University, 6th of Oc-tober City 12566, Egypt;
* Correspondence: ahmed.adel@pharma.cu.edu.eg (A.A.H.); ghada.ah.fawzy@pharma.cu.edu.eg (G.A.F.)

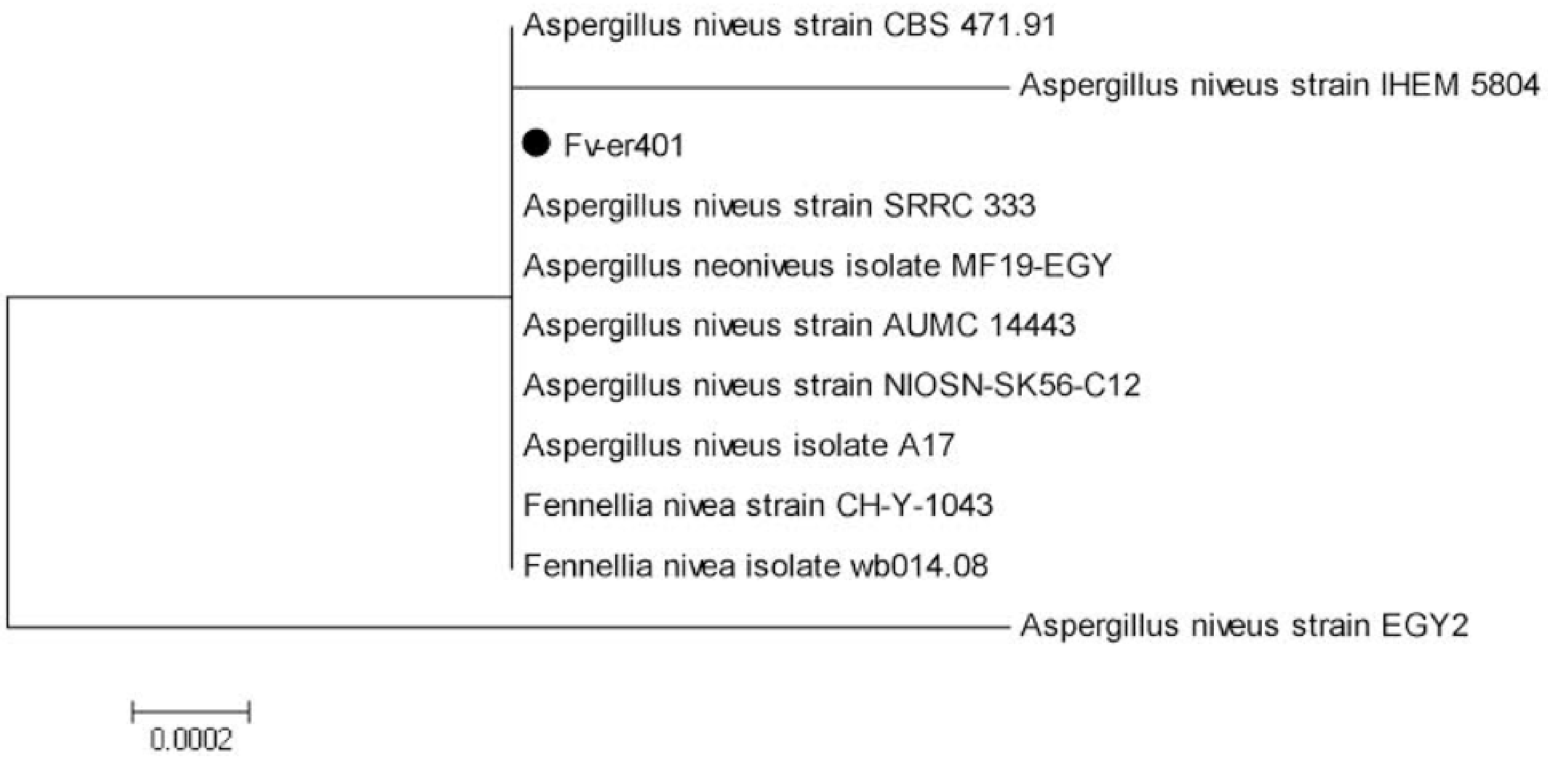

Figure S1. Phylogenetic analysis of *Aspergillus niveus* Fv-er401.

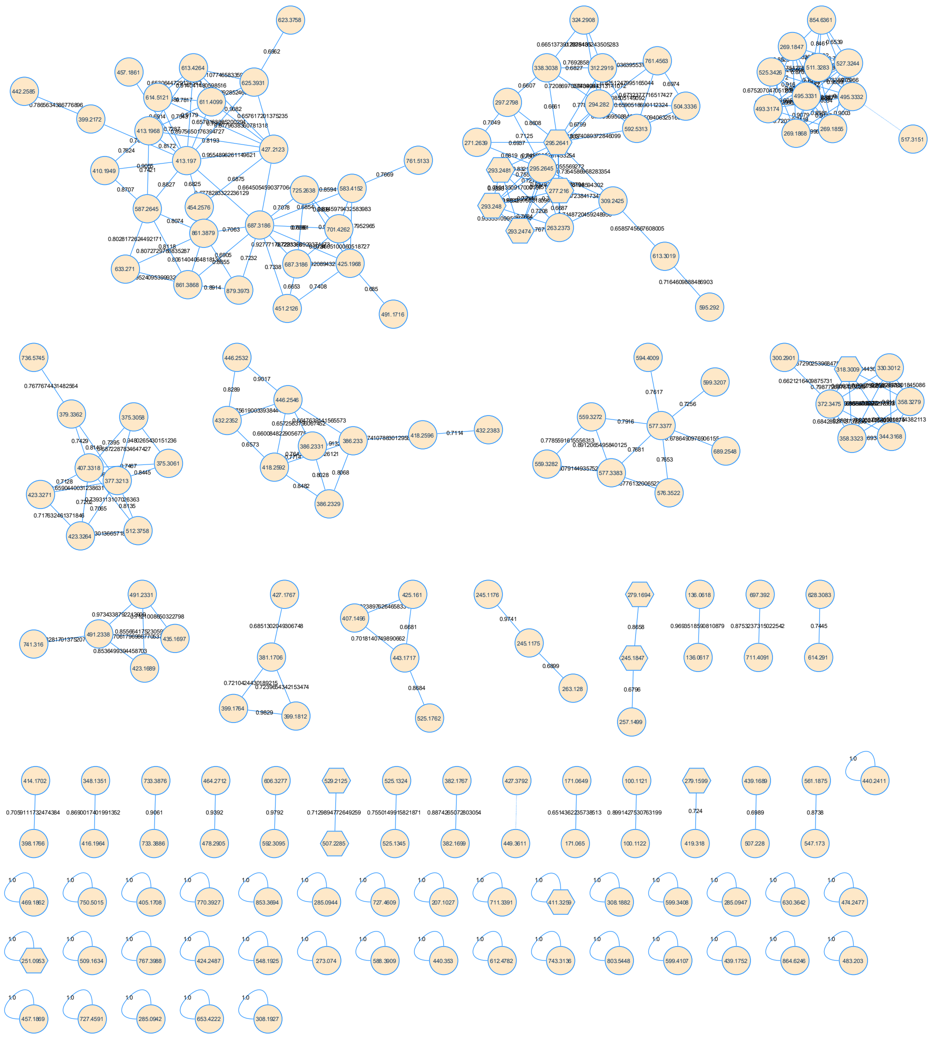

Figure S2. GNPS molecular network of *Aspergillus niveus* Fv-er401 ethyl acetate extract in the positive ion mode. The node label represents precursor mass (*m/z*), while the edge label represents the cosine score.

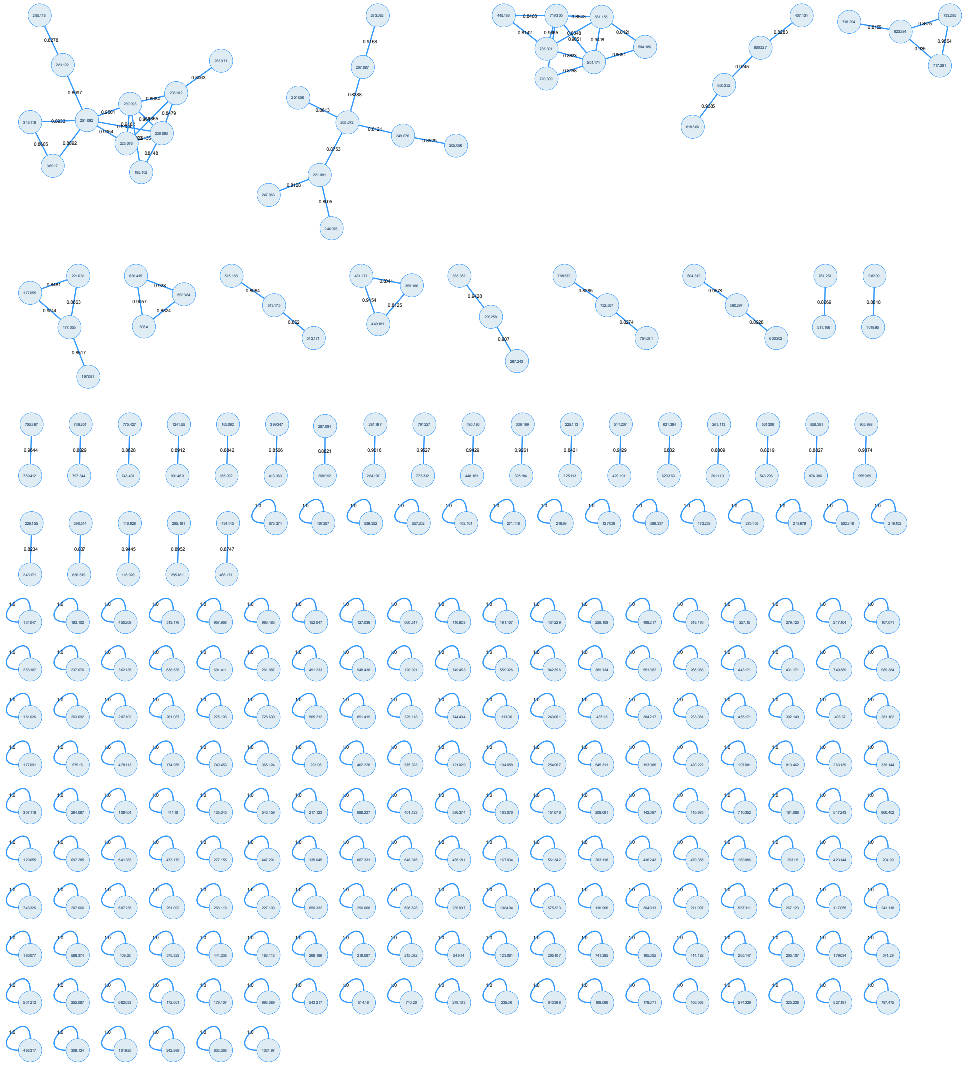

Figure S3. GNPS molecular network of *Aspergillus niveus* Fv-er401 ethyl acetate extract in the negative ion mode. The node label represents precursor mass (*m/z*), while the edge label represents the cosine score.

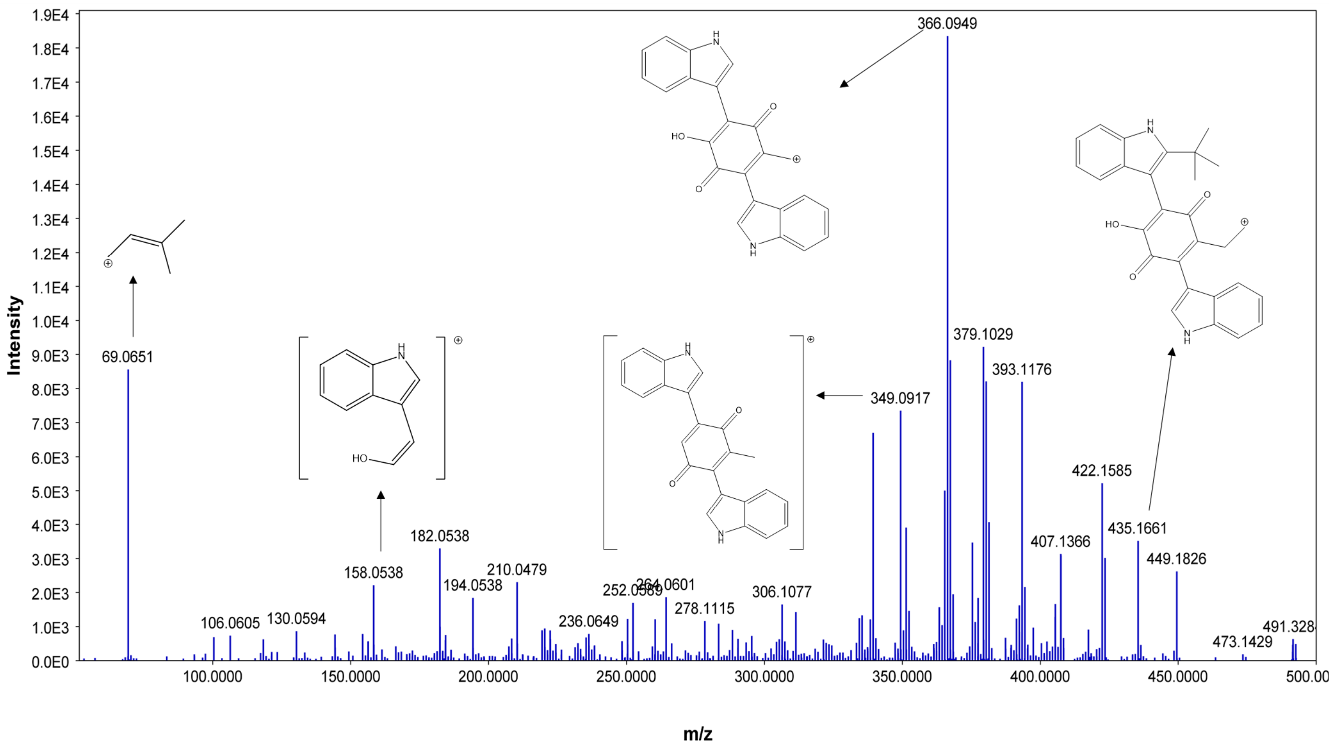

Figure S4. MS/MS fragmentation of terrequinone A (Compound 7).

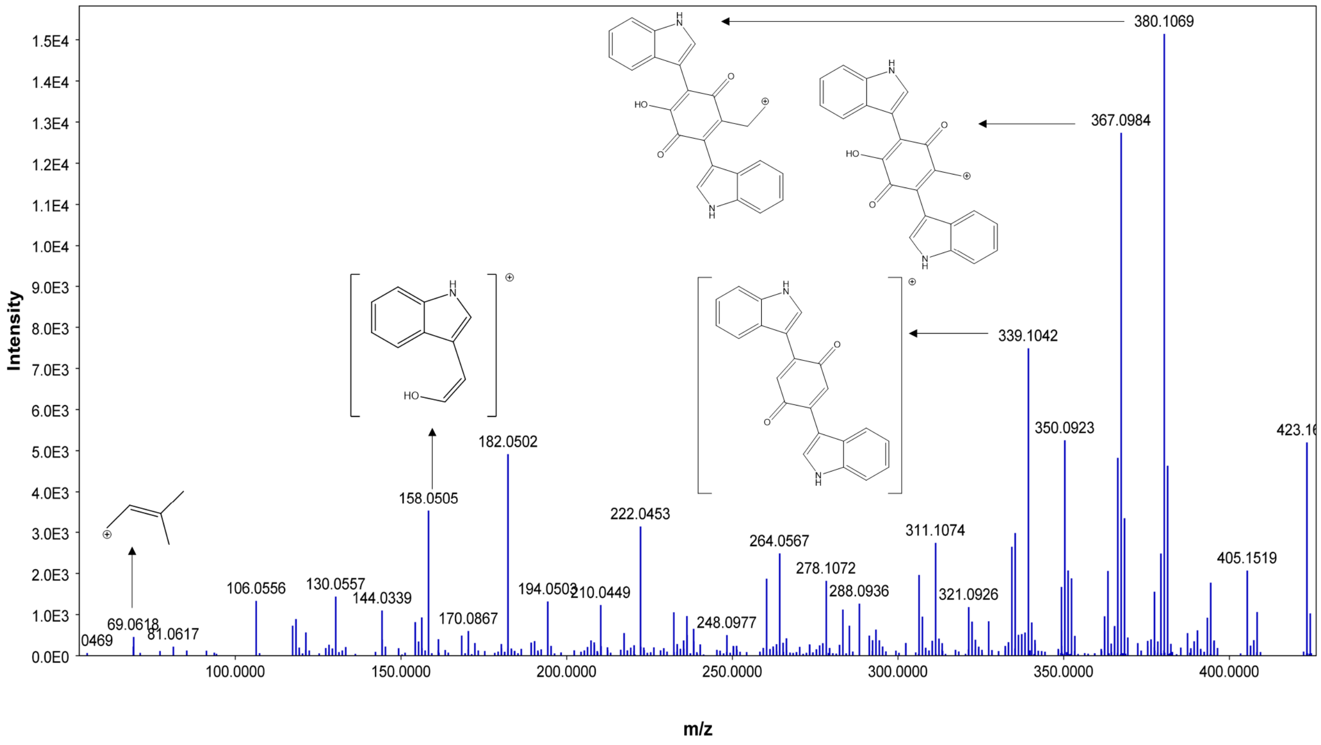

Figure S5. MS/MS fragmentation of ochrindole D (Compound 8).

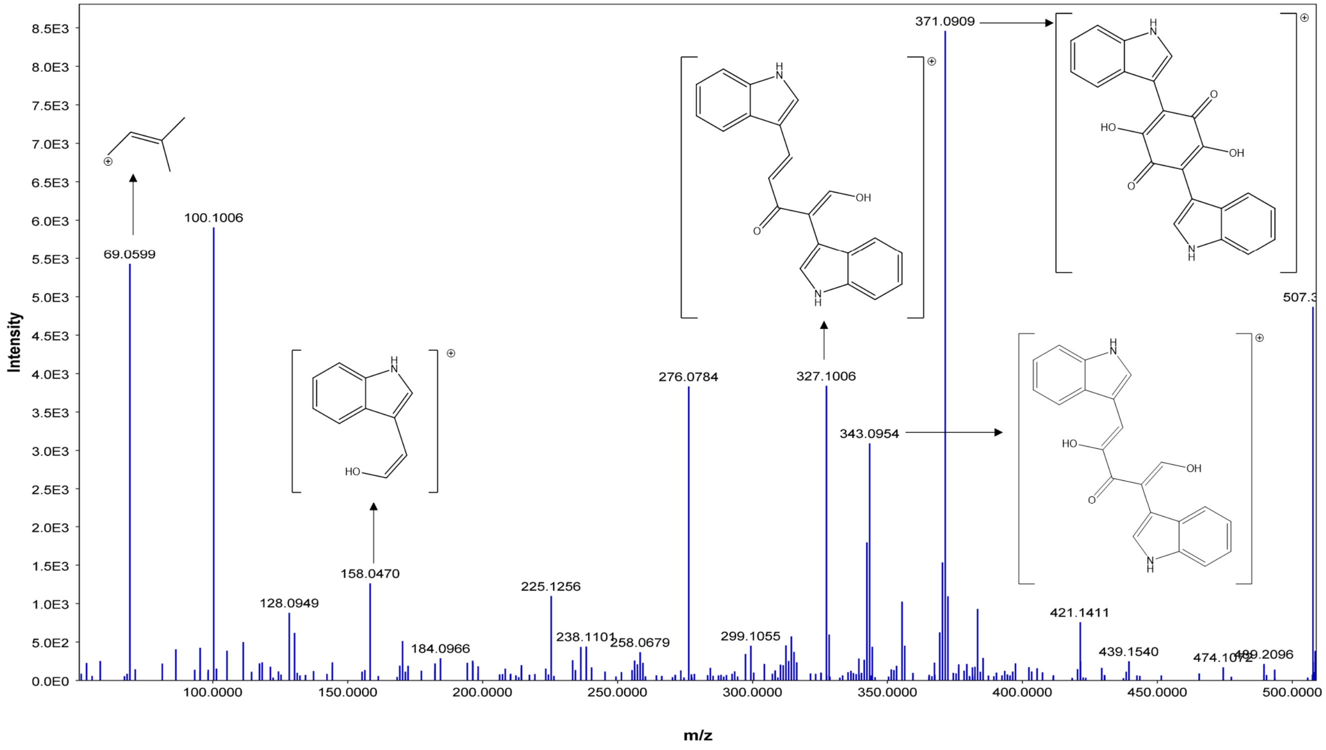

Figure S6. MS/MS fragmentation of asterriquinone CT5 (Compound 9).

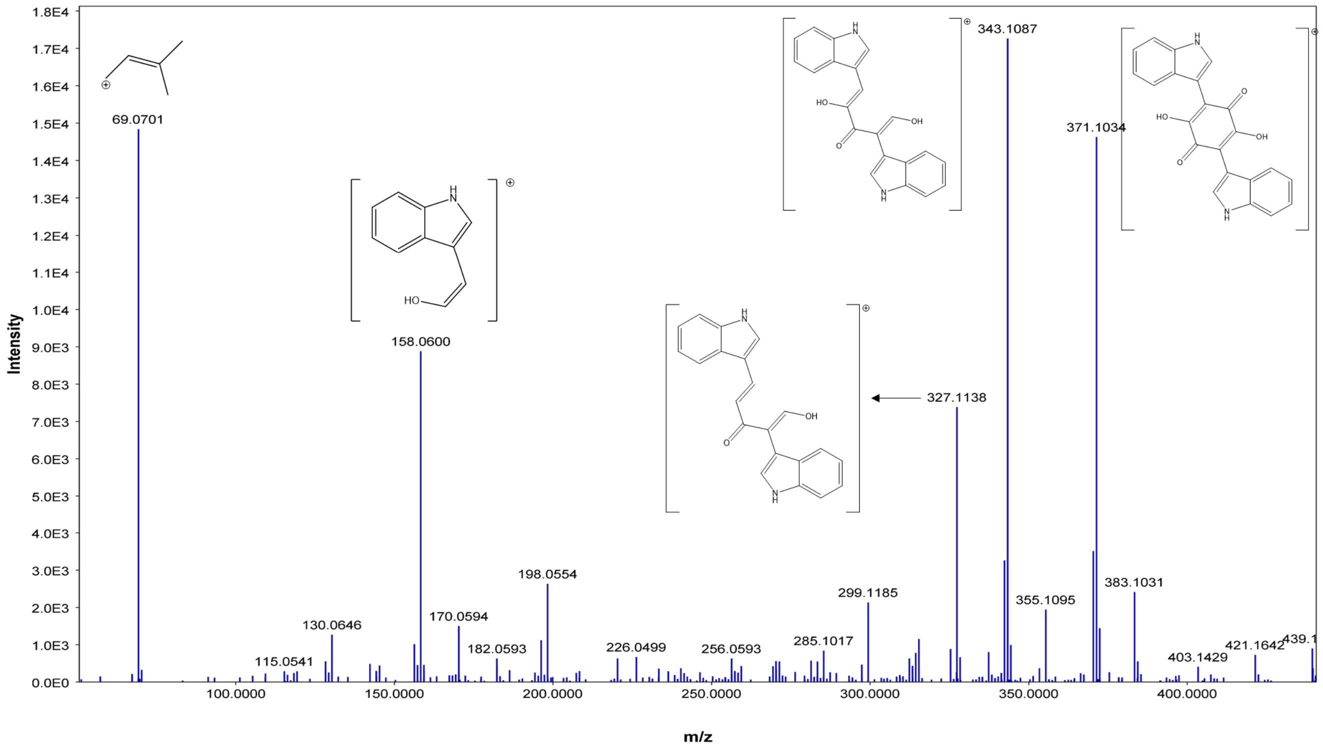

Figure S7. Predicted fragmentation pattern of Asterriquinone SU-5228 (Compound 6).

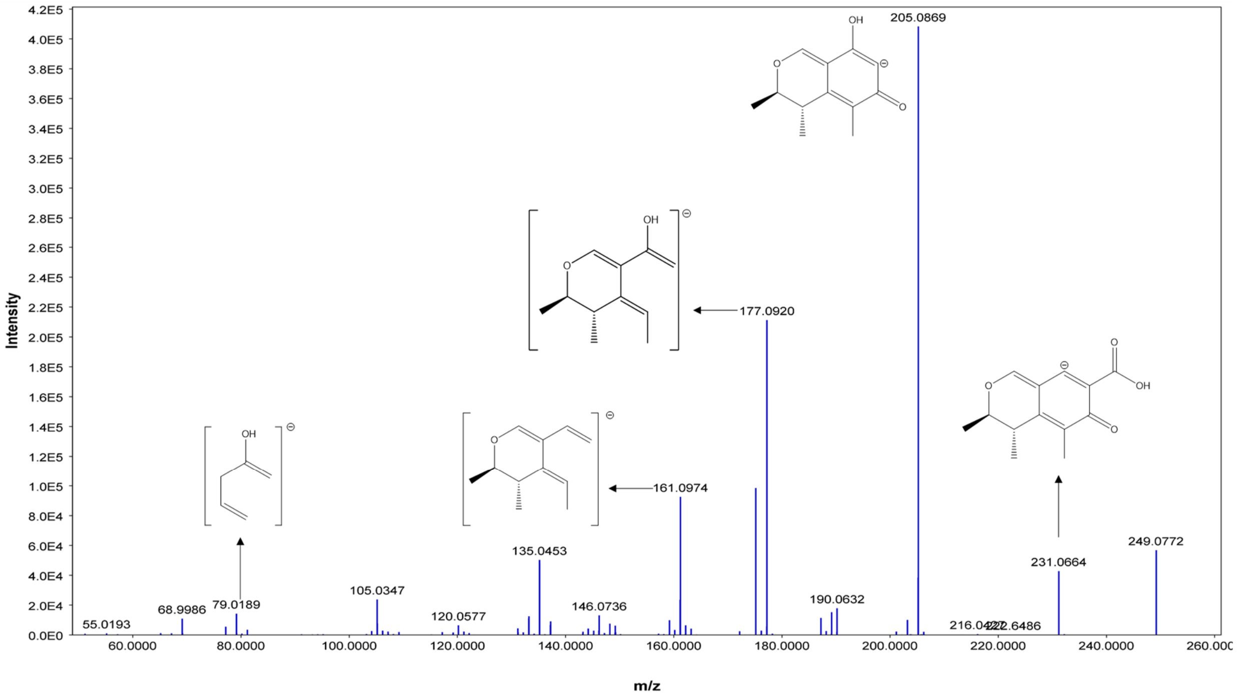

Figure S8. MS/MS fragmentation of citrinin (Compound 3).

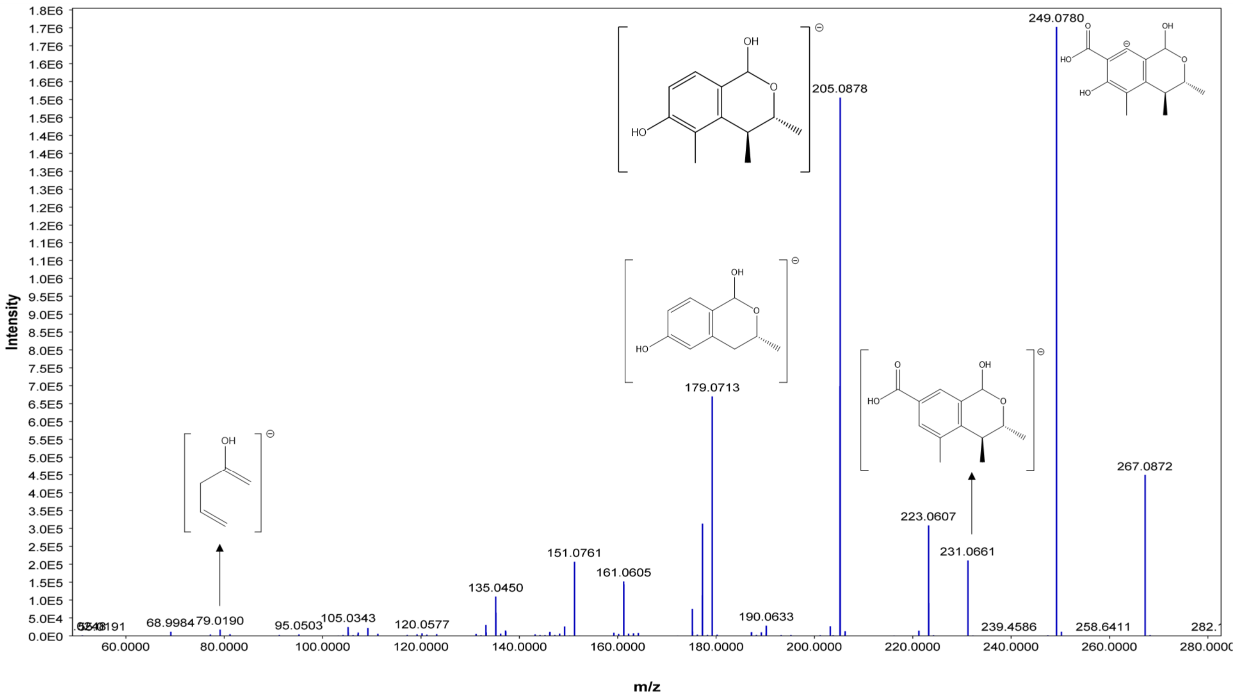

Figure S9. MS/MS fragmentation of citrinin hydrate (Compound 2).

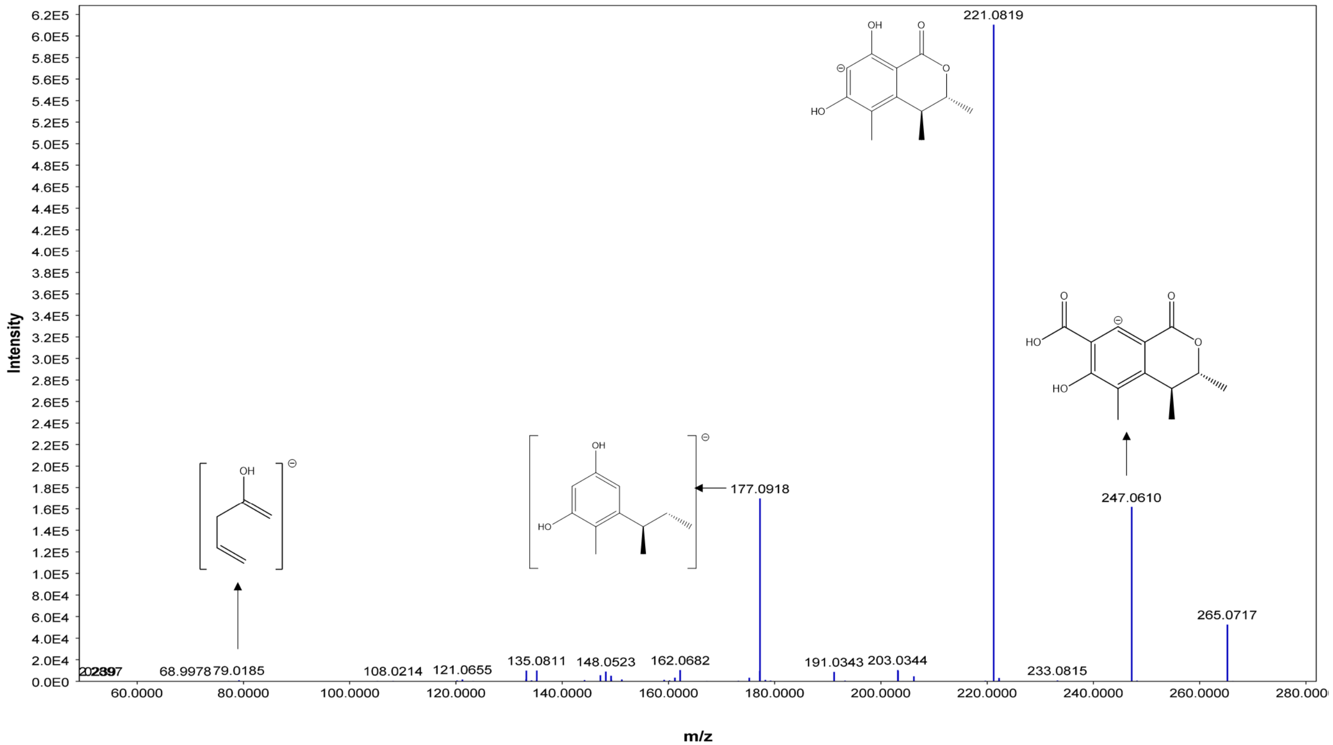

Figure S10. MS/MS fragmentation of dihydrocitrinone (DHC, Compound 1).

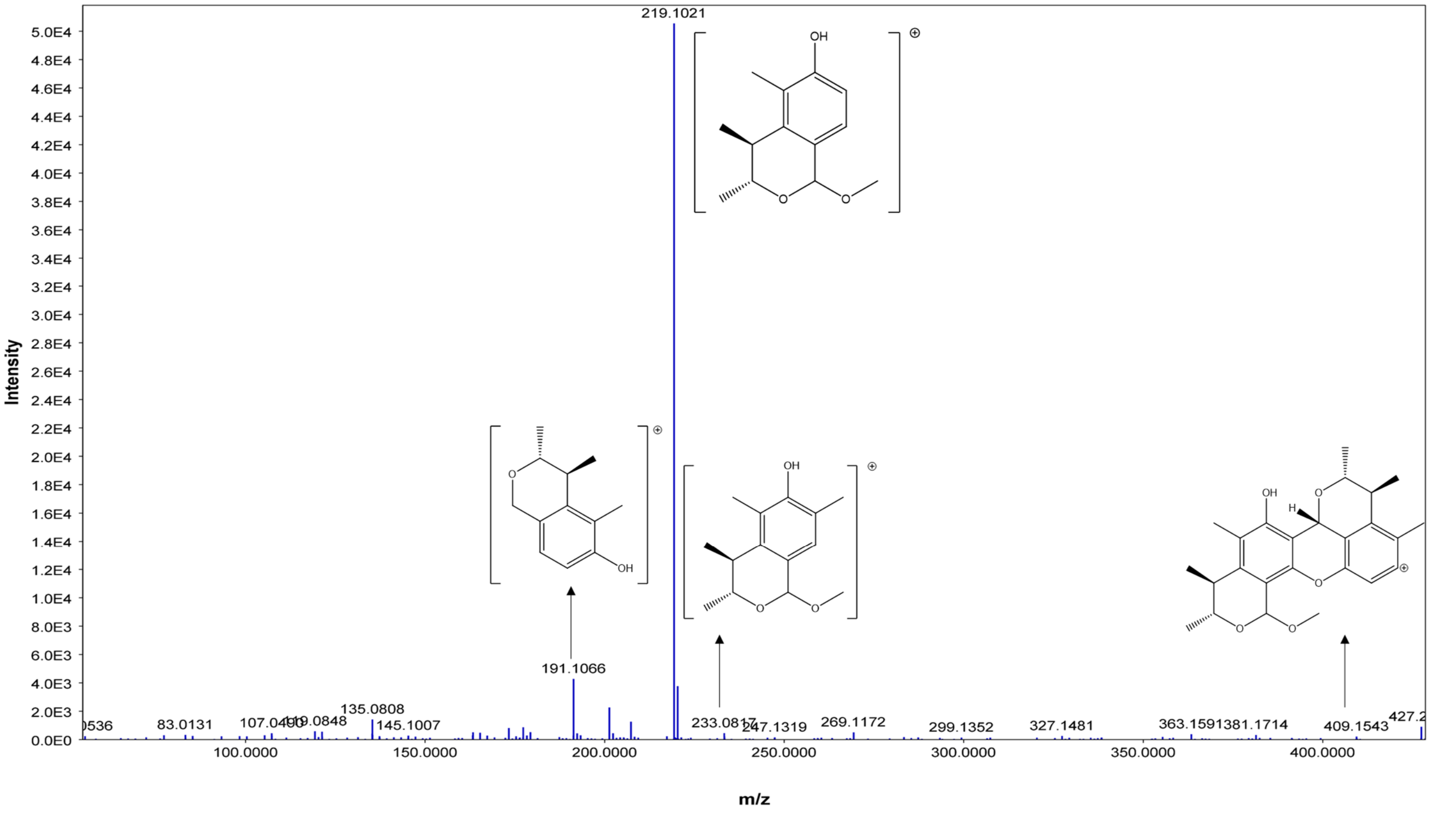

Figure S11. MS/MS fragmentation of dicitrinol A (Compound 4).

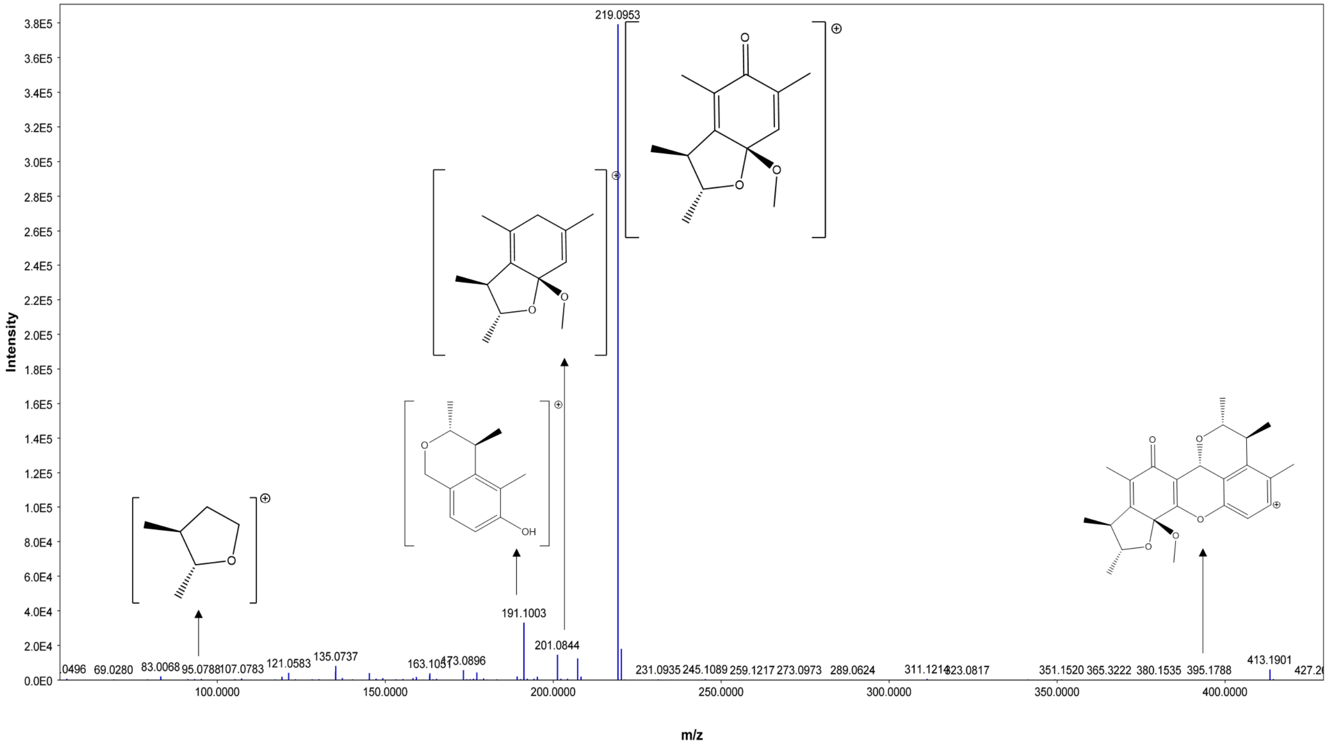

Figure S12. MS/MS fragmentation of penicitrinol B (Compound 5).

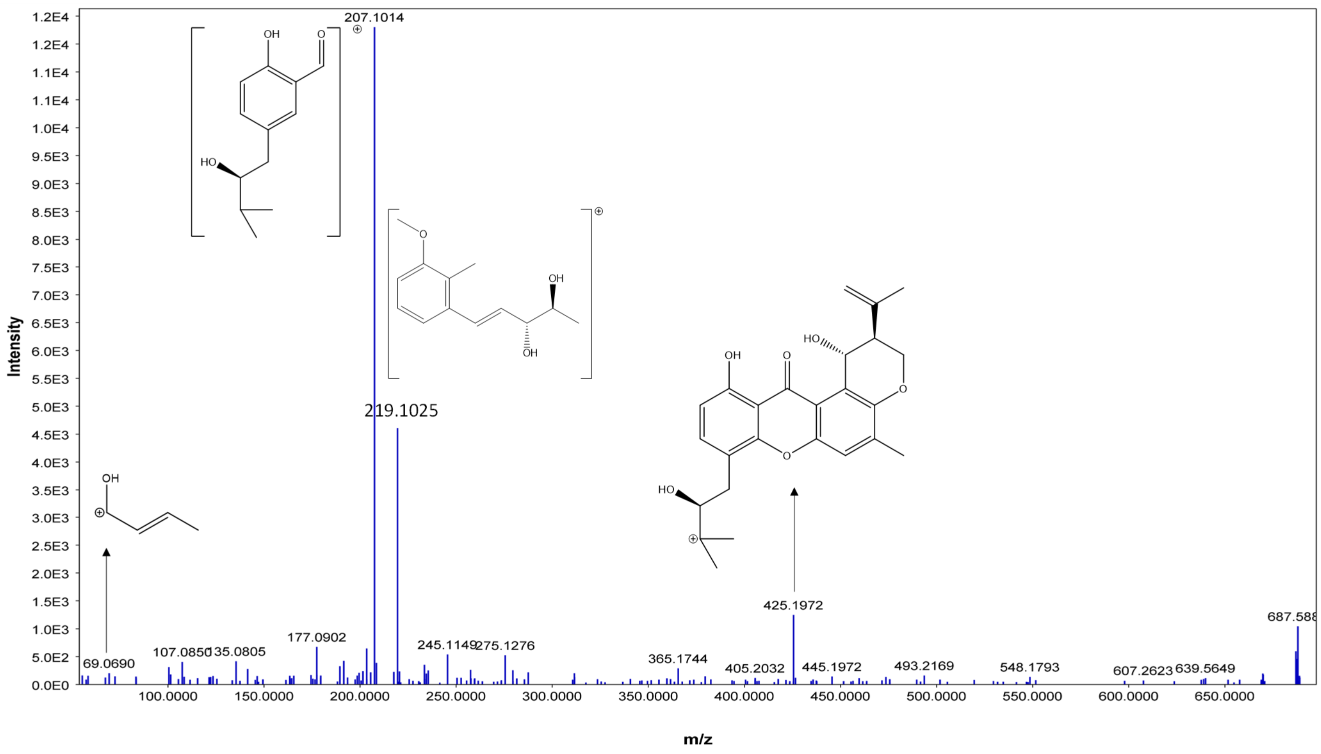

Figure S13. MS/MS fragmentation of varioxiranol G (Compound 10).

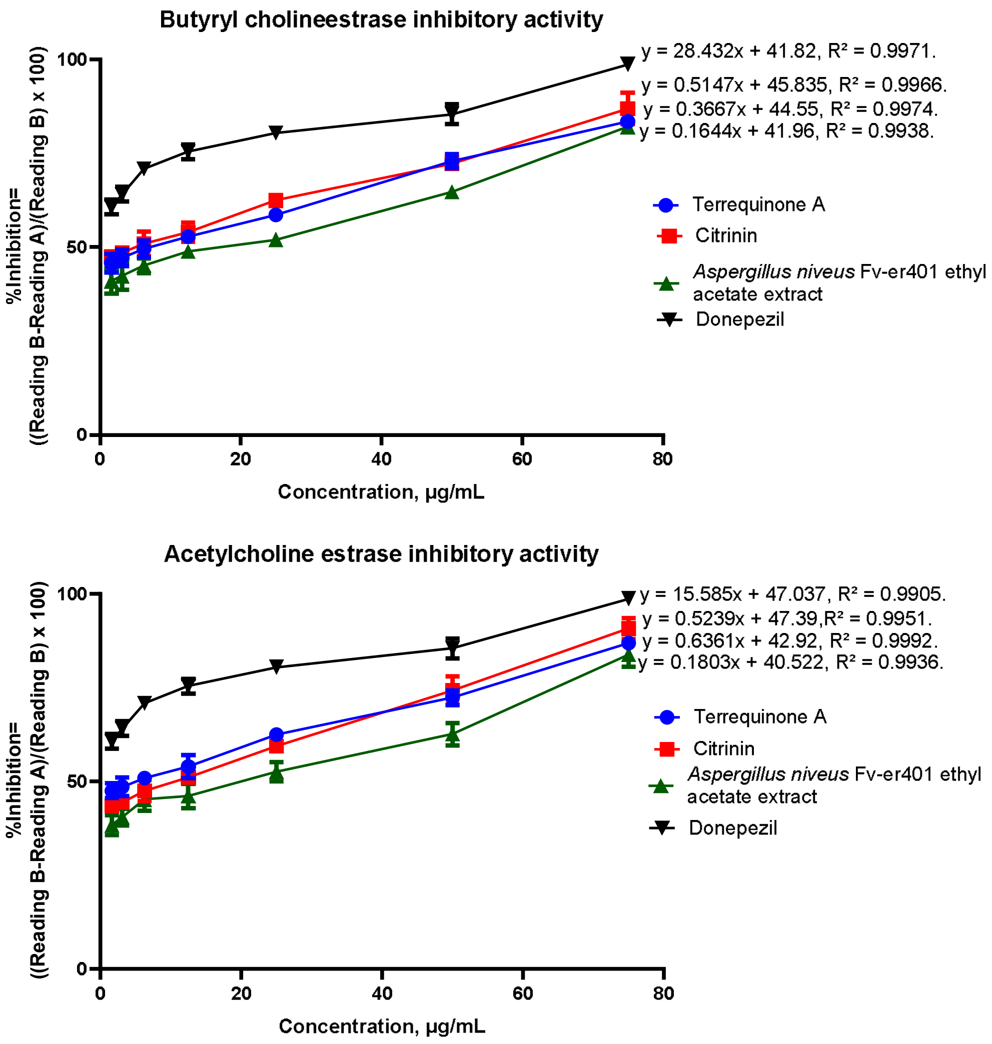

Figure S14. Graphs of acetylcholinesterase and butyrylcholinesterase inhibitory activity. The intensity of the developed color after the addition of inhibitor was measured at 405 nm as Reading A, and a control without the inhibitor was measured as Reading B. IC50 values (concentration at which there is 50 % enzyme-catalyzed reaction) were calculated from the equation.

Table S1. Putatively identified features of *Aspergillus niveus* Fv-er401 ethyl acetate extract in positive and negative modes.

No.	R_t (min)	Compound name	Adduct	Precursor mass	Molecular formula	MS/MS Fragmentation Product Ions	Chemical class
1	1.0519	Ile-leu	M+H	245.1847	$C_{12}H_{24}N_2O_3$	69.0671, 86.0933, 87.0961, 110.0556	Peptide
2	2.1895	5-Amino-2-[[(*Z*)-3-methylhex-2-enoyl]amino]-5-oxopentanoic acid	M+H	257.1499	$C_{12}H_{20}N_2O_4$	69.0701, 84.0444, 86.0964, 129.0657	Carboxamide
3	2.6321	**Huaspenone B**	M-H	267.1235	$C_{14}H_{20}O_5$	85.0661, 123.0816, 167.0713, 179.1077	Polyketides
4	2.6968	2-Hydroxy-3-(3,4,5-trimethoxyphenyl)propanoic acid	M-H	255.0872	$C_{12}H_{16}O_6$	152.0477, 167.0711, 193.0502, 255.0868	Organic acid
5	2.7436	*N*,*N*,2-Trimethylallylamin	M+H	100.1122	$C_6H_{13}N$	56.0402, 58.0558, 71.9185, 100.1005	Amines
6	2.7984	Phenol A acid	M-H	239.0967	$C_{12}H_{16}O_5$	109.0658, 151.077, 177.0556, 239.0924	Organic acid
7	2.8687	2,2-Dimethyl-6-methoxy-4-chromanone	M+H	207.1027	$C_{12}H_{14}O_3$	143.0857, 174.0679, 189.0914, 207.102	Chromanone
8	3.0662	3-Acetyl-5-(5-hydroxyhexa-1,3-dien-1-yl)-4-methoxy-5-methyl-2,5-Dihydrofuran-2-one	M-H	265.1079	$C_{14}H_{18}O_5$	121.0656, 123.0815, 167.0702, 179.1072	Furanone
9	3.2509	**Phomalone**	M-H	253.1072	$C_{13}H_{18}O_5$	109.0659, 151.076, 177.0551, 195.066	Phomalone
10	3.2701	**Dihydrocitrinone**	M-H	265.0733	C13H14O6	79.0185, 162.0682, 177.0918, 221.0819, 247.0610	Benzopyran
11	3.5003	**4,6-Dimethylcurvulinic acid**	M-H	237.0767	$C_{12}H_{14}O_5$	178.0632, 193.0872, 219.0661, 237.0759	Phenolic acid
12	3.5003	**Globosumone C**	M-H	283.0827	$C_{13}H_{16}O_7$	178.0635, 193.0869, 219.0663, 237.0767	Benzoate ester
13	3.6026	**JG002CPB**	M+H	592.3095	$C_{32}H_{41}N_5O_6$	134.0888, 169.1258, 70.0591, 197.1203	Peptides

14	3.685	**7-Hydroxyresorcylide**	M-H	307.1187	$C_{16}H_{20}O_6$	57.0349, 205.0862, 249.0768, 307.1184	Resorcylide derivatives
15	3.7497	**Diclavatol**	M-H	343.1186	$C_{19}H_{20}O_6$	255.1025, 281.0812, 325.1076, 343.118	Dimethylated tetraketide
16	3.7689	**Cycloaspeptide C**	M+H	628.3083	$C_{35}H_{41}N_5O_6$	368.1917, 437.2483, 495.2182, 600.313	Peptides
17	3.7689	**JG002CPA**	M+H	606.3277	$C_{33}H_{43}N_5O_6$	70.058, 134.0877, 183.14, 211.135	Peptides
18	3.9906	**Norlobaric acid**	M+H	443.1717	$C_{24}H_{26}O_8$	407.1384, 408.1416, 425.1492, 426.1524	Depsidones
19	4.0729	**Citrinin hydrate**	M-H	267.0909	$C_{13}H_{16}O_6$	79.0190, 179.0714, 205.0881, 249.0783, 267.0874	Benzopyran
20	4.1291	**Aspergillicin D**	M+H	697.392	$C_{36}H_{52}N_6O_8$	70.0652, 86.0963, 98.0599, 126.0544	Peptides
21	4.166	**Citrinin**	M-H	249.0802	$C_{13}H_{14}O_5$	79.0189, 161.0974, 177.0920, 205.0859, 231.0664	Benzopyran
22	4.1711	**Folipastatin**	M+H	381.1706	$C_{23}H_{24}O_5$	339.123, 348.1359, 351.1235, 363.1598	Depsidone
23	4.1845	**Deflectin 2a**	M+H	399.2172	$C_{24}H_{30}O_5$	83.0411, 177.1176, 205.1129, 207.0917	Polyketides
24	4.2126	**Aspochalasin S**	M+Na	424.2487	$C_{24}H_{35}NO_4$	376.1913, 391.2137, 406.2377, 425.1954	Cytochalasan alkaloids
25	4.2584	**Aspergillicin C**	M+H	711.4091	$C_{37}H_{54}N_6O_8$	70.0653, 98.0601, 126.0548, 134.0963	Peptides
26	4.4246	**Epi-aszonalenin A**	M+H	416.1964	$C_{25}H_{25}N_3O_3$	69.0609, 130.0541, 147.0443, 306.1132	Peptide alkaloids
27	4.4612	**Phytosphingosine**	M+H	318.3009	$C_{18}H_{39}NO_3$	56.0408, 57.0609, 60.0354, 282.2682	Sphingoid base
28	4.4801	**Linoleoyl ethanolamide**	M+H	324.2908	$C_{20}H_{37}NO_2$	60.0447, 74.0603, 93.0697, 306.2794	*N*-acylethanolamine
29	4.5355	**Aspergisidone**	M+H	425.161	$C_{24}H_{24}O_7$	351.0865, 389.1392, 407.1503, 408.1537	Depsidones
30	4.554	**Palmitoyl serinol**	M+H-H_2O	312.2919	$C_{19}H_{39}NO_3$	55.045, 69.0596, 86.0491, 95.0743	Fatty Acyls

31	4.5717	**Terretonin C**	M-H	429.1919	$C_{24}H_{30}O_7$	81.034, 205.0858, 249.0759, 429.1906	Polyketides
32	4.5724	Erythro-sphingosine	M+H	300.2901	$C_{18}H_{37}NO_2$	55.0544, 56.0498, 67.0542, 69.0701	Sphingosine
33	4.6925	2-Amino-9-methyl-4-octadecene-1,3,8-triol	M+H	330.3012	$C_{19}H_{39}NO_3$	56.0499, 60.0449, 74.0602, 282.28	Sphingosine
34	4.7756	*N*-acetylsphinganine	M+H	344.3168	$C_{20}H_{41}NO_3$	56.0499, 60.0448, 88.076, 282.2801	Sphinganine
35	4.8499	**Aspochalasin T**	M+H	418.2596	$C_{24}H_{35}NO_5$	71.0493, 88.0759, 148.0969, 271.1696	Cytochalasan alkaloids
36	5.0435	**2-Amino-9-methylicos-4-ene-1,3,8-triol**	M+H	358.3323	$C_{21}H_{43}NO_3$	56.0458, 57.0661, 60.0405, 74.056	Dehydrophytosphingosine
37	5.062	**Demethyltrichodimerol**	M+H	483.203	$C_{27}H_{30}O_8$	215.0976, 233.1079, 234.1102, 251.0823	Oxidized dimers of sorbicillin
38	5.0989	N-(1,3-dihydroxyicosan-2-yl) acetamide	M+H	372.3475	$C_{22}H_{45}NO_3$	56.0498, 60.0448, 88.0757, 310.3109	Acetamide
39	5.0989	**Dicitrinol A**	M+H	427.2123	$C_{25}H_{30}O_6$	191.1066, 201.0906, 219.1021, 220.1052, 233.0817, 409.1543	Polyketide
40	5.1543	**Penicitrinol B**	M+H	413.197	$C_{24}H_{28}O_6$	95.0788, 191.1003, 201.0844, 219.0953, 220.0988, 395.1788	Polyketide
41	5.2098	**Asterriquinone SU-5228**	M+H	439.1689	$C_{27}H_{22}N_2O_4$	69.0701, 158.06, 327.1138, 343.1087, 371.1034	Bisindole alkaloid
42	5.2652	**JBIR-82**	M+H	495.3332	$C_{29}H_{42}N_4O_3$	72.081, 100.1121, 101.1154, 114.0914	Terpeptin analogs
43	5.3757	**Asperphenamate**	M+Na	529.2125	$C_{32}H_{30}N_2O_4$	100.112, 117.07, 238.123, 292.096	Carboxylic ester
44	5.764	**Terrequinone A**	M+H	491.2331	$C_{32}H_{30}N_2O_3$	69.0651, 158.0538, 349.0917, 366.0949, 379.1029, 435.1661	Bisindole alkaloid
45	5.764	**Ochrindole D**	M+H	423.1689	$C_{27}H_{22}N_2O_3$	69.0618, 158.0505, 339.1042, 350.0923, 367.0984, 380.1069	Bisindole alkaloid
46	5.8194	Phophatidylethanolamine(15:0/22:6)	M+H	750.5015	$C_{42}H_{72}NO_8P$	57.0703, 59.0493, 71.0857, 117.0542	Lipids
47	5.9579	**Asterriquinone CT5**	M+H	507.228	$C_{32}H_{30}N_2O_4$	69.0599, 158.0470, 327.1006, 343.0954, 371.0909	Bisindole alkaloid

48	6.0503	**Gaidic acid**	M+Na	277.216	$C_{16}H_{30}O_2$	67.0517, 79.0516, 81.0306, 93.0667	Trihydroxybenzoic acid
49	6.2904	**Ergosterol peroxide**	M+H	411.3259	$C_{28}H_{44}O_3$	69.0693, 173.0949, 175.1107, 191.1055	Steroid derivative
50	6.4567	Methyl linolenate	M+H	293.248	$C_{19}H_{32}O_2$	67.0467, 81.0618, 95.077, 109.092	Fatty acid
51	6.4567	**Cytosporolide B**	M+H	559.3272	$C_{32}H_{46}O_8$	233.0804, 234.084, 251.091, 329.1746	Sesquiterpenes
52	6.5675	**Varioxiranol G**	M+H	687.3186	$C_{40}H_{46}O_{10}$	69.0690, 207.1024, 219.1022, 425.1973, 493.2238	Polyketides
53	6.8723	3,4,7,12,16-Pentamethyloctadeca-1,3,5,7,9,11,13,15,17-nonaene-1,18-diyl) bis(6-hydroxy-2,4,4-trimethylcyclohex-2-en-1-one)	M+H	611.4099	$C_{41}H_{54}O_4$	69.0695,191.1058,219.1017,220.1047	Fatty alcohol
54	6.9277	Methyl palmitate	M+H	271.2639	$C_{17}H_{34}O_2$	55.0549, 57.0705, 69.0704, 71.0857	Fatty acid
55	7.0109	Methyl oleate	M+H	297.2798	$C_{19}H_{36}O_2$	55.0545, 57.0701, 69.0701, 83.0855	Fatty acid
56	7.094	Diadinoxanthin	M+H	583.4152	$C_{40}H_{54}O_3$	189.0918, 207.1023, 208.1059, 273.0741	Phytopigment
57	7.1771	2'-Methoxy-3,1'-Dihydroxy-Β,Ψ-Caroten-4-One	M+H	613.4264	$C_{41}H_{56}O_4$	69.0795, 191.1184, 219.1134, 220.1168	Carotenoids
58	7.3157	3-Hydroxyergosta-4,6,8(14),22-tetraen-15-on	M+H	423.3271	$C_{29}H_{42}O_2$	57.0704, 69.0701, 83.0855, 85.0652	Steroids

Table S2: Types and compositions of media

Medium	Composition
Potato dextrose broth	Potato extract (4.0 g), and dextrose (20.0 g), distilled water to 1000mL, pH 5.6
Potato dextrose agar	Potato extract (4.0 g), dextrose (20.0 g), and agar (15.0 g) distilled water to 1000mL. pH 5.6
Wickerham agar	Yeast extract (3.0 g), malt extract (3.0 g), peptone (5.0 g), dextrose (10.0 g), and agar (20.0 g), distilled water to 1000mL. pH 7.3

Table S3: Parameters for MZmine processing of UHPLC-MS/MS data

Processing step	Parameter	Selected values
Mass list	MS1 noise level	1.0E3
	MS2 noise level	1.0E2
	Rt (retention time in minutes)	1-7.5 min
Chromatogram building	Algorithm	ADAP chromatogram builder
	Min group size in number of scans	5
	Group intensity threshold	3.0E3
	Min highest intensity	3.0E3
	m/z tolerance	0-20 ppm
Deconvolution	Algorithm	Local minimal search
	Chromatographic threshold	30.0%
	Search minimum in RT range	0.2 min
	Minimum relative height	10 %
	Minimum absolute height	3.0E3
	Min ratio of peak top/edge	1
	Peak duration range	0-2 min
Isotope grouping	*m/z* tolerance	0-10 ppm
	RT tolerance	0.2 min
	Maximum charge	3
Alignment	Algorithm	Join aligner
	m/z tolerance	0-20 ppm
	Weight for *m/z*	75%
	RT tolerance	0.2 min
	Weight for RT	25%

Table S4. 2D, 3D interactions, and receptor pocket positioning for donepezil and the docked co-crystallized inhibitors within the binding sites of both AChE and BuChE receptors.

Comp.	R	2D interactions	3D interactions	3D positioning
**Donepezil**	AChE	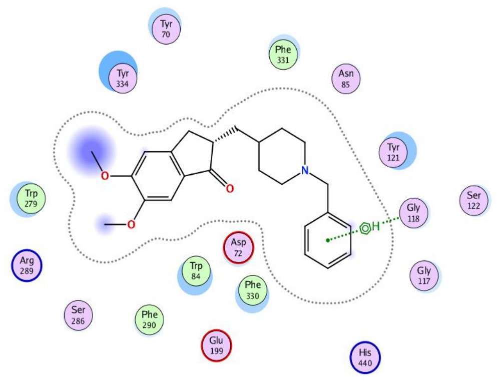	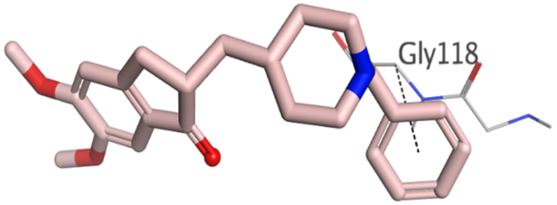	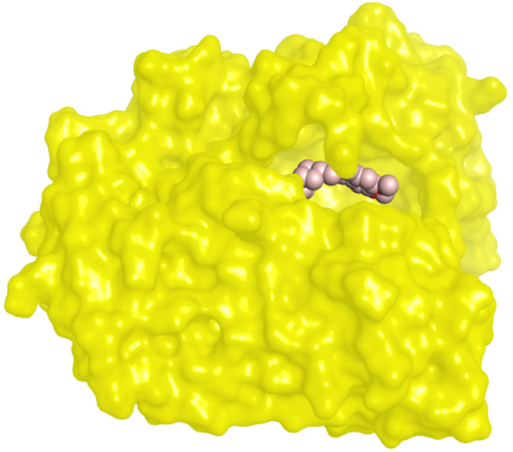
	BuChE	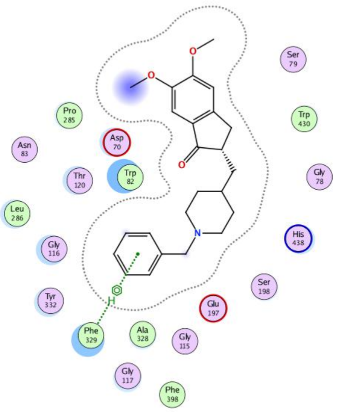	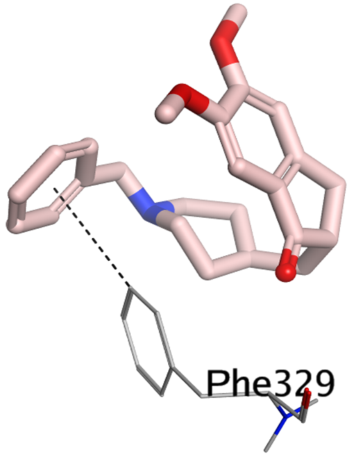	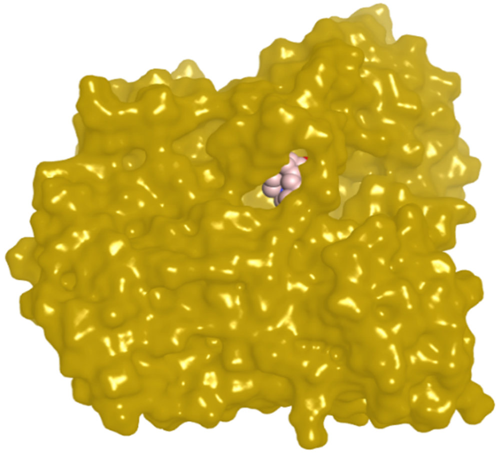

Comp.	R	2D interactions	3D interactions	3D positioning
**Co-crystallized inhibitor**	AChE	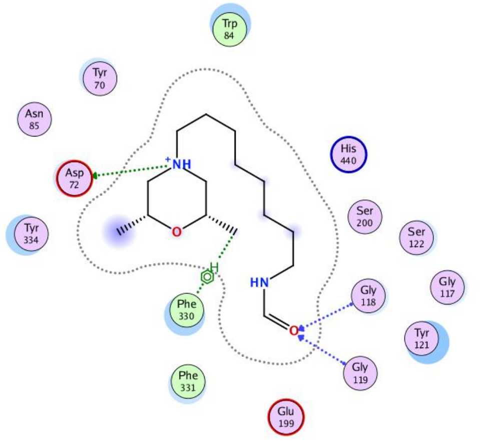	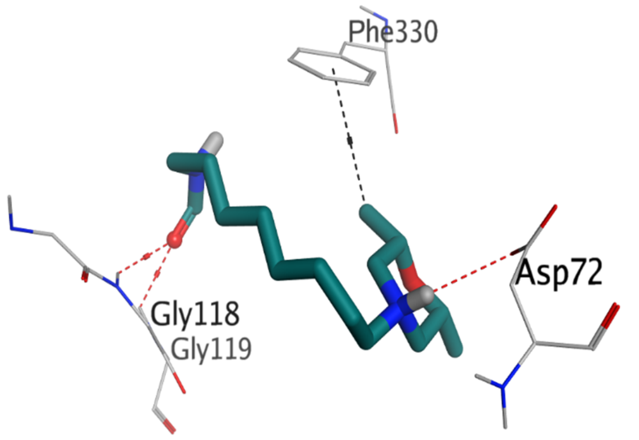	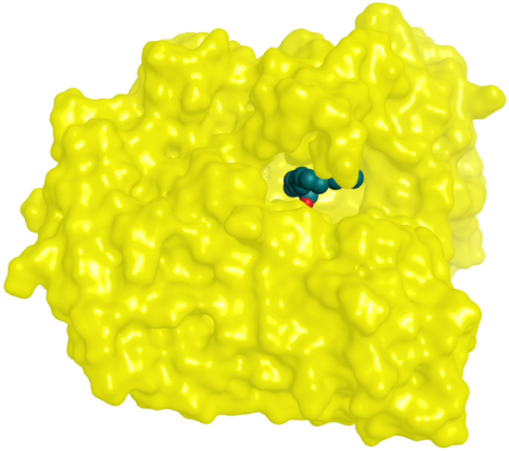
	BuChE	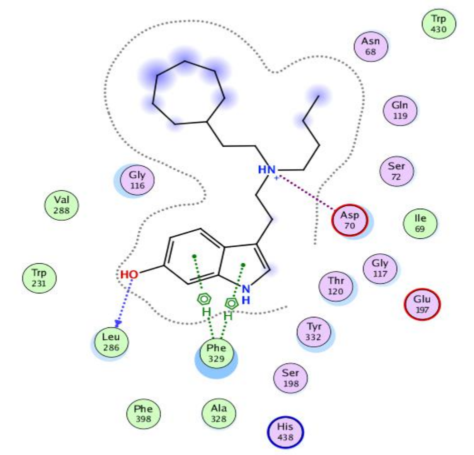	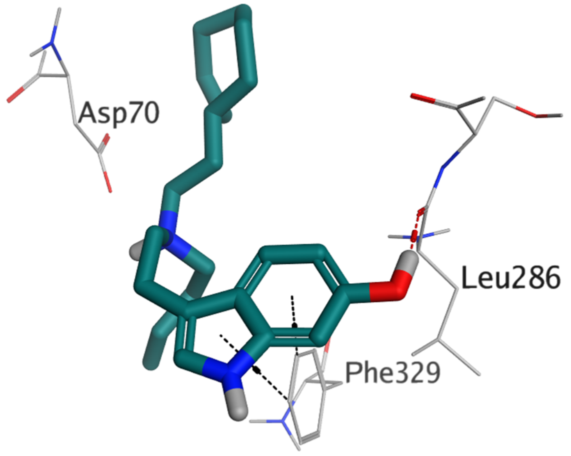	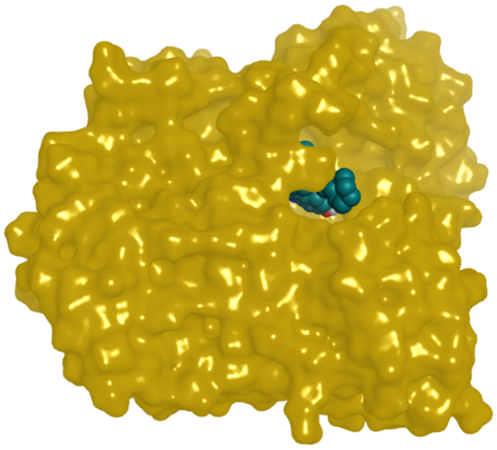

R*: Receptor.
